# Integration of synaptic phototransistors and quantum dot light-emitting diodes for visualization and recognition of UV patterns

**DOI:** 10.1126/sciadv.abq3101

**Published:** 2022-10-12

**Authors:** Hyojin Seung, Changsoon Choi, Dong Chan Kim, Ji Su Kim, Jeong Hyun Kim, Junhee Kim, Soo Ik Park, Jung Ah Lim, Jiwoong Yang, Moon Kee Choi, Taeghwan Hyeon, Dae-Hyeong Kim

**Affiliations:** ^1^ Center for Nanoparticle Research, Institute for Basic Science (IBS), Seoul 08826, Republic of Korea.; ^2^ School of Chemical and Biological Engineering, Institute of Chemical Processes, Seoul National University, Seoul 08826, Republic of Korea.; ^3^ Center for Opto-Electronic Materials and Devices, Post-silicon Semiconductor Institute, Korea Institute of Science and Technology (KIST), Seoul 02792, Republic of Korea.; ^4^ Department of Energy Science and Engineering, Daegu Gyeongbuk Institute of Science and Technology (DGIST), Daegu 42988, Republic of Korea.; ^5^ Department of Materials Science and Engineering, Center for Future Semiconductor Technology (FUST), Ulsan National Institute of Science and Technology (UNIST), Ulsan 44919, Republic of Korea.; ^6^ Department of Materials Science and Engineering, Seoul National University, Seoul 08826, Republic of Korea.

## Abstract

Synaptic photodetectors exhibit photon-triggered synaptic plasticity, which thus can improve the image recognition rate by enhancing the image contrast. However, still, the visualization and recognition of invisible ultraviolet (UV) patterns are challenging, owing to intense background noise. Here, inspired by all-or-none potentiation of synapse, we develop an integrated device of synaptic phototransistors (SPTrs) and quantum dot light-emitting diodes (QLEDs), facilitating noise reduction and visualization of UV patterns through on-device preprocessing. The SPTrs convert noisy UV inputs into a weighted photocurrent, which is applied to the QLEDs as a voltage input through an external current-voltage–converting circuit. The threshold switching characteristics of the QLEDs result in amplified current and visible illumination by the suprathreshold input voltage or nearly zero current and no visible illumination by the input voltage below the threshold. The preprocessing of image data with the SPTr-QLED can amplify the image contrast, which is helpful for high-accuracy image recognition.

## Teaser

The integrated device of synaptic phototransistors and QLEDs facilitates visualization and recognition of UV patterns.

## INTRODUCTION

Photodetectors with persistent photoconductivity (PPC) (*1*, *2*) exhibit optoelectronic memory effects (*3*, *4*). The photocurrent generation of these photodetectors (*5*–*7*) shows similar features to the short-term plasticity (STP) and long-term potentiation (LTP) in human synapse (*8*). The photocurrent is weighted in proportion to the amount of light irradiation, i.e., the duration and intensity of irradiation. These photodetectors are called synaptic photodetectors (SPDs) (*4*, *9*, *10*). Because of the photon-triggered synaptic plasticity (*11*–*14*), SPDs can perform in-sensor preprocessing of the acquired image data during the front-end image-sensing step (*15*–*18*), producing a preprocessed image with reduced background noise and enhanced contrast (*3*, *19*–*21*). The preprocessed image can be recognized with a higher accuracy than the noisy original image (*22*, *23*). Therefore, in comparison with conventional photodetectors (*24*–*26*) and image-processing devices (*27*–*29*) (fig. S1A), which have computational burdens for these preprocessing steps via software-based approaches, SPDs provide a hardware-based alternative for image recognition with higher efficiency (i.e., less computational burden).

Ultraviolet (UV) imaging has been used for various real-life applications, such as collection of forensic evidence (*30*), detection of hazardous gas leakages (*31*), and measurement of skin abnormalities (*32*), providing useful optical information that humans cannot see with bare eyes. However, UV light is easily absorbed and/or scattered by objects, and thus, the raw UV information reflected on the target object contains a large amount of noise (*33*). In this regard, the SPD has an advantage in UV imaging, owing to its in-sensor preprocessing, which can reduce the background noise without additional computations (fig. S1B). Amorphous indium gallium zinc oxide (a-IGZO) is a promising material for the fabrication of the UV-responsive synaptic phototransistor (SPTr) because of its wide bandgap (*34*–*36*), PPC induced by ionization of oxygen vacancy (*1*), and relatively high mobility (*37*, *38*). However, because of the high noise level of the original UV image data, the residual background noise often remains even after the conventional in-sensor preprocessing step. Therefore, a novel UV imaging system is demanded to achieve high-accuracy image recognition with minimum image filtering during the back-end processing step (*39*–*41*). Besides, visualization strategies of the preprocessed UV images for human vision are required.

Here, we report an integrated device of SPTrs and quantum dot light-emitting diodes (QLEDs) for visualization and recognition of UV patterns through signal-or-none (SoN) on-device preprocessing (fig. S1C). The integrated SPTr-QLED is inspired by the all-or-none potentiation of human synapse (*42*). The photocurrent generated by the SPTrs linearly depends on the duration and intensity of UV irradiation, which promotes UV-triggered synaptic plasticity and in-sensor preprocessing. The weighted photocurrents from the SPTrs are used to drive the QLEDs. The QLEDs with threshold switching characteristics enable the nonlinear filtering of the preprocessed signal from the SPTrs for SoN on-device preprocessing, which leads to an amplified signal output with reduced background noise. At the same time, the QLED visualizes the preprocessed images to reveal the acquired UV information for human vision. Furthermore, we validate the effectiveness of SoN on-device preprocessing through simulations that include image generation and recognition. The deep neural network can recognize the SoN on-device preprocessed images with higher accuracy (>86%) than the images acquired by conventional image sensors (<36%) or the preprocessed images by conventional SPDs (<50%).

## RESULTS

### SoN type signal acquisition and preprocessing

**Fig. 1.**
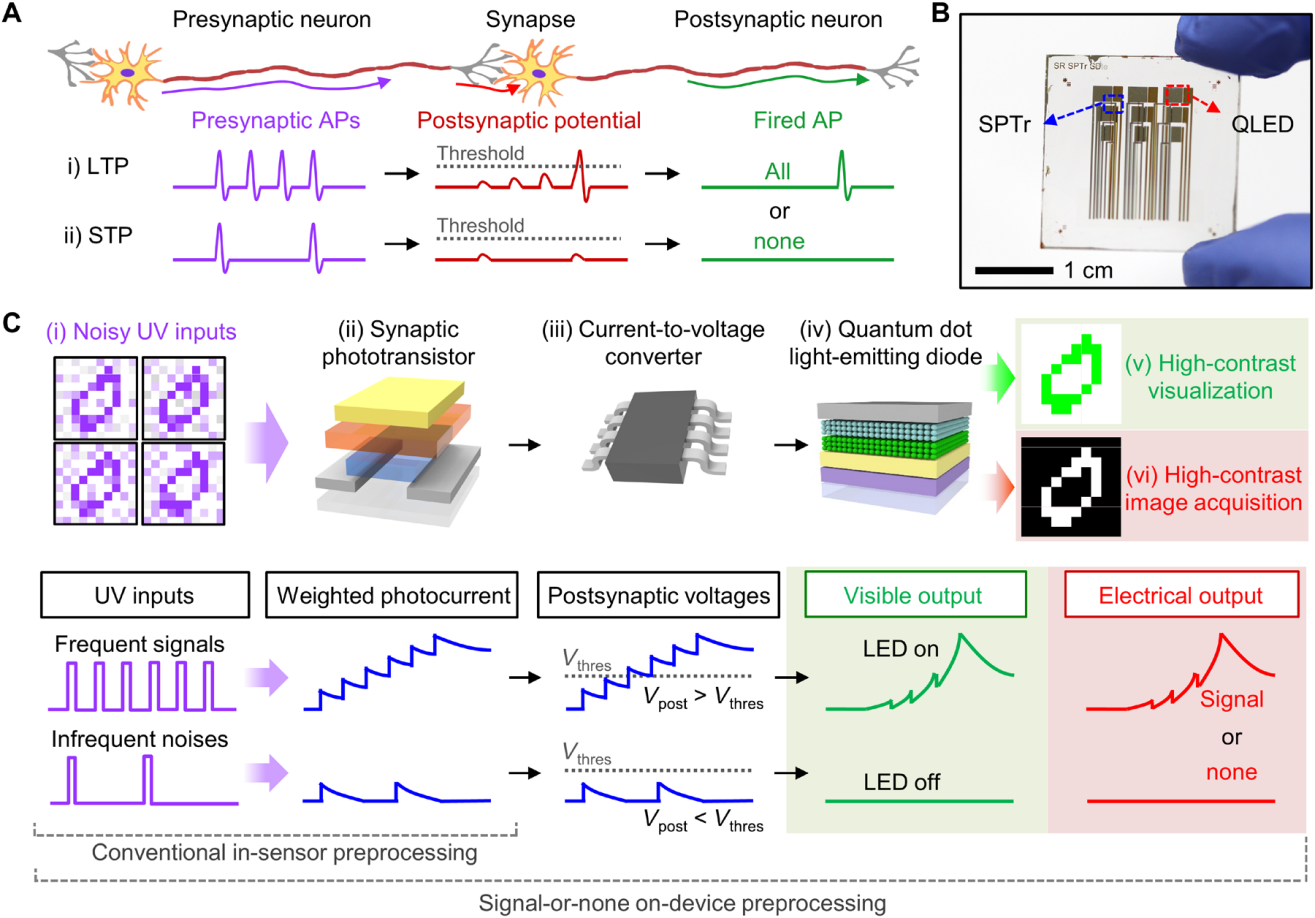
Integration of SPTrs and QLEDs for visualization and recognition of UV patterns. (**A**) Schematic illustration of human synapse and its neural signal transmission mechanism. The human synapse features synaptic plasticity and all-or-none potentiation. (**B**) Photograph of an integrated device of the SPTr array (located at the top side of a glass substrate) and the QLED array (located at the bottom side of a glass substrate). (**C**) Schematic illustrations showing the process for deriving the high-contrast visible and electrical outputs from the noisy UV patterns by using the integrated SPTr-QLED (top). The bottom schematic diagrams show the signals at each step for the frequent signals (top) and infrequent noise (bottom).

The synapse, a fundamental building block of neural networks, plays an important role in the transmission of neural signals (Fig. 1A) (*43*). Neural signal transmission begins with the release of neurotransmitters induced by the firing of a presynaptic neuron. The neurotransmitters are bound to the receptors of the postsynaptic neuron, generating a postsynaptic potential. The amount of the neurotransmitter release is dependent on the strength of synaptic connection (i.e., synaptic weight). A temporal enhancement of the synaptic weight induces a small amount of neurotransmitter releases, leading to a low postsynaptic potential (*8*, *44*). Such a small enhancement quickly decays to the initial state (i.e., STP). In contrast, repetitive presynaptic action potentials (APs) cause a relatively permanent enhancement of the synaptic weight, resulting in a large amount of neurotransmitter release and a high postsynaptic potential [i.e., LTP (*45*)]. Such strengthening/weakening of the synaptic weight caused by presynaptic APs is called synaptic plasticity (*8*, *44*). Meanwhile, the firing of postsynaptic neurons depends on the summated amplitude of the postsynaptic potentials. When the temporally or spatially summed postsynaptic potentials in the postsynaptic neuron exceed a threshold, the postsynaptic neuron fires an AP. In contrast, when the postsynaptic potential remains low, no AP is fired. Such a postsynaptic AP firing mechanism is called as all-or-none potentiation of the synapse (*42*, *46*).

An integrated device for UV imaging and visualization, the SPTr-QLED (Fig. 1B), exhibits the photoresponse inspired by synaptic plasticity and all-or-none potentiation of the human synapse. Such photoresponse can be used for nonlinear noise filtering of noisy UV information (i.e., SoN on-device preprocessing), and the QLED can visualize the preprocessed image. In particular, the none state (i.e., no AP firing) inspires the subthreshold noise filtering in SoN on-device preprocessing (*42*, *46*). The SPTrs generate a weighted photocurrent in response to sequential noisy UV inputs (Fig. 1C, i and ii), a combination of frequent/strong signals and infrequent/weak noises (fig. S2 and section S1) (*3*). The frequent signals generate a large photocurrent, while the infrequent noises lead to a small photocurrent. Even after this background noise reduction, which is comparable to the in-sensor preprocessing of the conventional SPDs, the background noise can still remain. The SPTr-QLED performs an additional on-device filtering step to reduce the residual noise from the in-sensor–preprocessed image. The current-to-voltage converters electrically connect the SPTrs and QLEDs to convert the weighted photocurrent (*I*_SPTr_) to the postsynaptic voltage (*V*_post_) (Fig. 1Ciii), and *V*_post_ is applied to the QLEDs as an input voltage (Fig. 1Civ). Because of the threshold-switching behavior of the QLED, the QLEDs are turned on only when *V*_post_ is over their threshold voltage (*V*_thres_). The QLEDs also show an exponential current increase in response to *V*_post_ over *V*_thres_, thereby producing the electrical and visible outputs with amplified contrast (Figs. 1C, v and vi). The QLEDs successfully function as a visualizer and a nonlinear filter, owing to their threshold-switching characteristics (*47*, *48*). Strong/frequent features of UV patterns could be extracted and visualized by the STPr-QLED without computationally expensive noise-removal steps used in conventional software-based preprocessing and filtering methods. Because the image recognition rate is degraded by the background noise, noise reduction down to a nearly zero level enables accurate image recognition by the deep neural network [e.g., ResNet50 (*49*)].

### Integrated device of SPTr and QLEDs

**Fig. 2.**
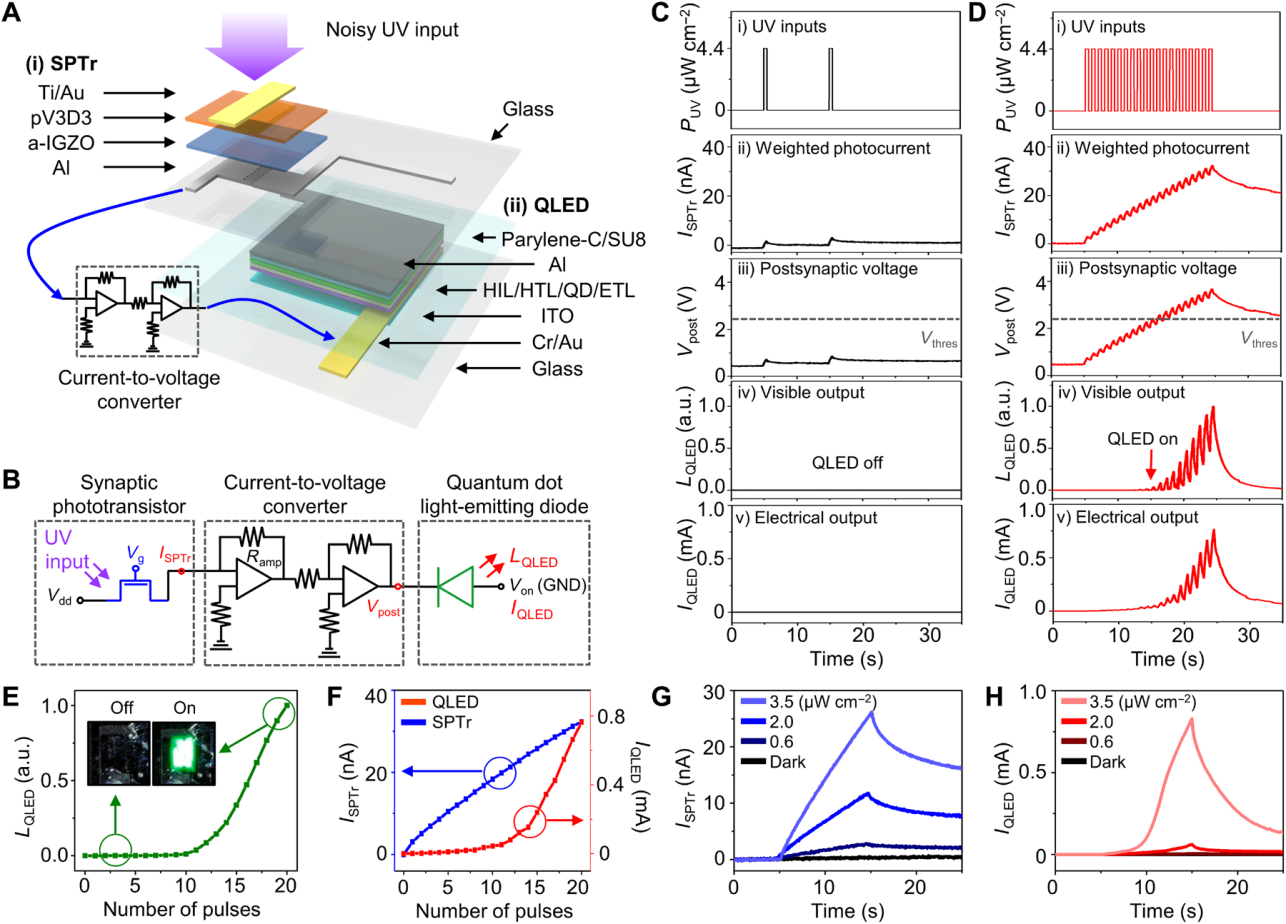
Integration of SPTr and QLED. (**A**) Exploded illustration of the SPTr-QLED. QD, quantum dot; ITO, indium tin oxide. HIL, hole injection layer. HTL, hole transporting layer. ETL, electron transporting layer. (**B**) Circuit diagram of the SPTr and QLED connected through the current-to-voltage converter. GND, ground. (**C** and **D**) Infrequent (C) or frequent (D) UV inputs applied to the SPTr-QLED (i) and the resultant device characteristics, e.g., the weighted photocurrent (ii), postsynaptic voltage (iii), visible output (iv), and electrical output (v), of the SPTr-QLED. (**E**) Luminance of the QLED over the pulse number. The insets show the photographs of the QLED turned off and on when the 1st pulse and the 20th pulse are irradiated, respectively. a.u., arbitrary units. (**F**) The photocurrents of the SPTr and QLED over the pulse number. (**G** and **H**) The photocurrents of the SPTr (G) and QLED (H) depending on the different UV intensities.

Figure 2A illustrates an individual SPTr-QLED pixel. The SPTr and QLED are assembled on the top and bottom sides of a glass substrate, respectively, and integrated through an external current-to-voltage converter (Fig. 2B). Photographs of the integrated SPTr-QLED and its fabrication process are described in fig. S3 and Materials and Methods. The SPTr consists of an a-IGZO channel and a poly(1,3,5-trimethyl-1,3,5-trivinyl cyclotrisiloxane) (pV3D3) dielectric layer. Because of the wide bandgap of a-IGZO (i.e., 3.6 eV), the SPTr generates photocurrent by the irradiation of UV light, while it shows negligible photoresponse to visible light (fig. S4). In addition, the SPTr features linearly time-dependent photocurrent generation and PPC under a negative gate bias (*V*_g,read_ = −4 V; fig. S5A), which leads to UV-triggered synaptic plasticity. The photocurrent can be reset by applying a positive gate bias (*V*_g,erase_ = 4 V) that returns the SPTr to its original state (after 35 s in fig. S5A). Furthermore, the SPTr is responsive to a wide range of light intensities (fig. S5B). The green QLED has a *V*_thres_ near 2.5 V, which is comparable to the bandgap divided by electron charge and shows an exponentially increasing current output under the suprathreshold bias conditions but a negligible current output under the subthreshold conditions (fig. S5C). Because of the double-layer encapsulation of Parylene-C and epoxy, QLEDs can be operated stably both under ambient condition and under UV irradiation (*50*). The optical microscope images and cross-sectional transmission electron microscope images of the SPTr and QLED are shown in fig. S6.

The weighted photocurrent (*I*_SPTr_) of the SPTr, *V*_post_ of the current-to-voltage converter, and the brightness (*L*_QLED_) and photocurrent (*I*_QLED_) of the QLED are measured for the infrequent and frequent UV inputs (*P*_UV_). The QLED derives no visible output and negligible electrical output by the infrequent UV inputs (two UV pulses with a duration of 0.5 s and a frequency of 0.1 Hz) (Fig. 2C). In contrast, by the irradiation of frequent UV inputs (Fig. 2Di; 20 UV pulses with a duration of 0.5 s and a frequency of 1.0 Hz), *I*_SPTr_ increases gradually as more UV inputs are applied (Fig. 2Dii). *V*_post_ also increases in proportion to *I*_SPTr_ and exceeds *V*_thres_ when the 10th UV pulse is applied (Fig. 2Diii). Then, the QLED emits visible light (Fig. 2Div), and thus, its luminance consistently increases (Fig. 2E). Photographs of the QLED before (QLED off) and after (QLED on) the irradiation of 20 pulses are shown in the insets in Fig. 2E. *I*_QLED_ also increases as more UV pulses are applied (Fig. 2Dv).

The key difference between *I*_SPTr_ and *I*_QLED_ is that *I*_QLED_ increases much more rapidly (exponential increase) than *I*_SPTr_ (linear increase) (Fig. 2F). Therefore, the ratio of photocurrents generated by frequent signals and infrequent noises of *I*_QLED_ (~362) is much larger than that of *I*_SPTr_ (~13) (fig. S7A). The *I*_SPTr_ and *I*_QLED_ show the linear and exponential photocurrent generation at various UV intensities, respectively, when continuous (10 s; Fig. 2, G and H) or pulsed (20 pulses; fig. S8) UV inputs are applied. The decaying characteristics of *I*_SPTr_ and *I*_QLED_ were also investigated. *I*_SPTr_ slowly decays with a long retention time (τ_SPTr_ >1000 s) due to PPC of the SPTr (fig. S5A). *I*_QLED_ decays more rapidly (τ_QLED_ ~2 s; calculated from Fig. 2Dv) because *I*_QLED_ has an exponentially amplified relationship to *I*_SPTr_. Therefore, the SPTr-QLED shows much less nonvolatile characteristics than the SPTr. However, because the residual *I*_QLED_ can affect the subsequent image acquisition and signal processing step, the SPTr-QLED needs to be reset to its initial state by applying a positive gate bias to the SPTr.

### Demonstration of SoN on-device preprocessing and visualization

**Fig. 3.**
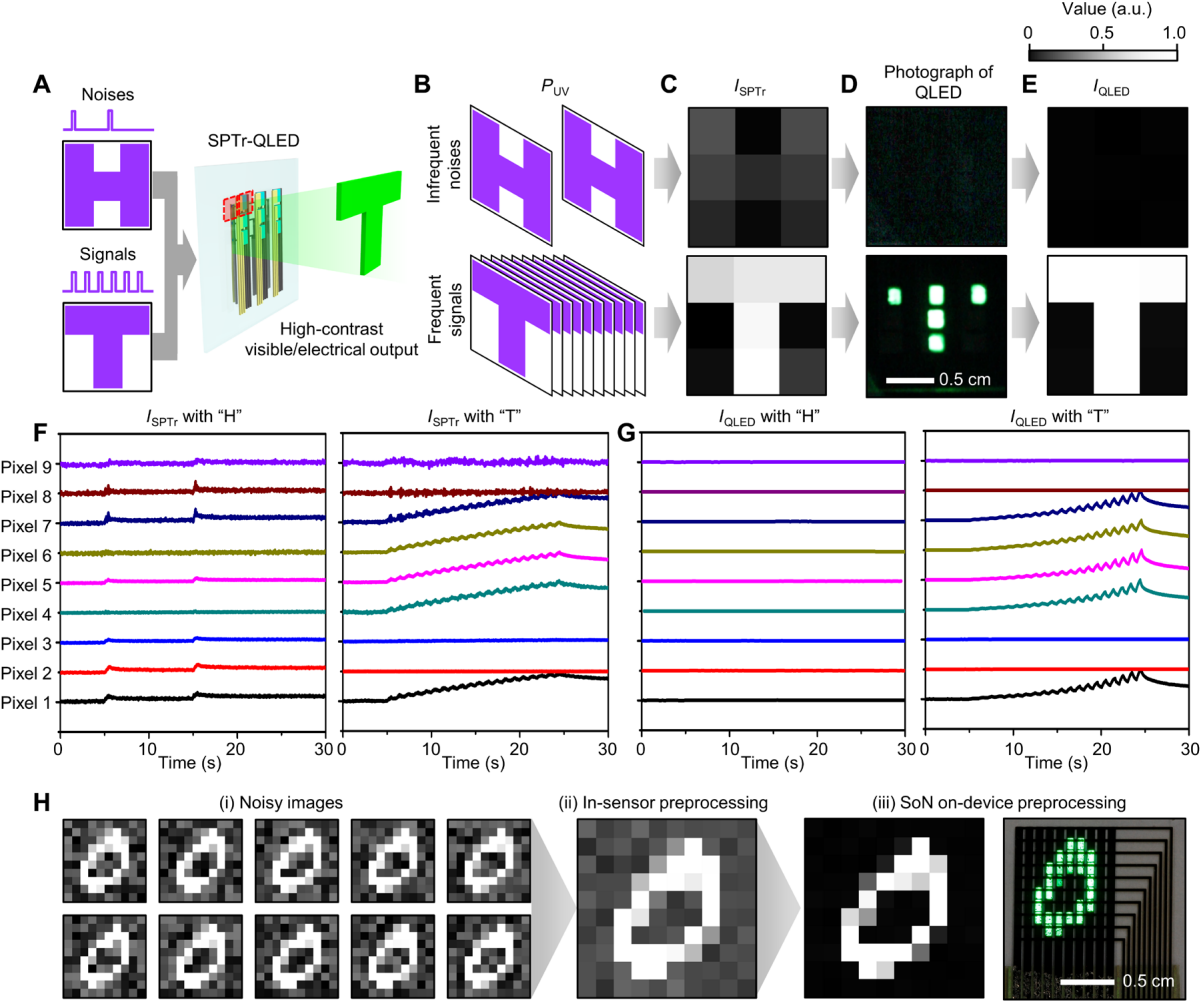
Array demonstration for visualization and acquisition of UV patterns. (**A**) Schematic illustrations for visualization and acquisition of UV patterns by the SPTr-QLED array. (**B**) UV inputs of letters H and T irradiated with different frequencies, each representing the infrequent noise and frequent signals. (**C** to **E**) The mapped *I*_SPTr_ (C), the photographs of QLED array (D), and the mapped *I*_QLED_ (E) after the SPTr-QLED array is irradiated with the UV patterns of H (top) and T (bottom). (**F** and **G**) *I*_SPTr_ (F) and *I*_QLED_ (G) of nine pixels of the array during the infrequent irradiation of H (left) and the frequent irradiation of T (right). (**H**) SoN on-device preprocessing of sequentially irradiated noisy images. (i) Ten noisy images of number 0, (ii) the in-sensor preprocessed image obtained by the conventional SPDs, (e.g., SPTr), and (iii) the SoN on-device preprocessed image (left) and a photograph of the 10 × 10 passive matrix QLED array visualizing the SoN on-device preprocessed image (right).

The photodetection, SoN on-device preprocessing, and visualization of UV patterns are demonstrated by using a 3 × 3 array of SPTr-QLEDs. Each SPTr and QLED in the array shows uniform characteristics, because the pixel-to-pixel variation in the transfer curves of SPTrs and the variation in the dark-/photocurrents of SPTrs and QLEDs are small (fig. S9). An infrequently irradiated UV pattern (letter “H”), corresponding to infrequent noises (two UV pulses with an intensity of 4.36 μW cm^−2^, a duration of 0.5 s, and a frequency of 0.1 Hz), and a frequently irradiated UV pattern (letter “T”), corresponding to frequent signals (20 UV pulses with an intensity of 4.36 μW cm^−2^, a duration of 0.5 s, and a frequency of 1.0 Hz), are projected onto the device (Fig. 3A). Shadow masks were used to create these UV noise or signal patterns. More details for the array demonstration are explained in fig. S10 and Materials and Methods.

According to the mapped *I*_SPTr_, a dim noise pattern of the letter H is still seen (Fig. 3C, top), although its intensity is much lower than the signal pattern of the letter T (Fig. 3C, bottom). This result indicates that the conventional in-sensor preprocessing by the SPTr only is insufficient to remove the background noise. In contrast, according to the SoN on-device preprocessing and its visulization result, the QLEDs show no visual output (Fig. 3D, top), and the mapped *I*_QLED_ is dark (Fig. 3E, top) with the infrequent UV inputs of “H,” while the QLEDs visualize the pattern T and produce a clear image of the letter T (Fig. 3, D and E, bottom) with the frequent UV inputs of “T.” The *I*_SPTr_ and *I*_QLED_ of nine pixels during the demonstrations are shown in Fig. 3 (F and G, respectively). Imaging and preprocessing of the frequent UV input of the letter T with lower UV intensities (2.0 and 2.8 μW cm^−2^) were demonstrated as well (fig. S11 and section S1). Movie S1 shows the visible output of the QLEDs during the demonstrations.

Another demonstration for a noisy environment was performed. Ten noisy UV images for the number “0” were generated by adding random noises (σ ~ 0.5) to the raw 0 image imported from Modified National Institute of Standards and Technology (MNIST) dataset, resized into a 10 × 10 shape (Fig. 3Hi). UV light that corresponds to the individual pixels of the noisy 10 × 10 images was sequentially irradiated to a unit device of the SPTr-QLED, by which all the pixels in the 10 × 10 images can be scanned one by one. This process was applied to every pixel of the 10 × 10 images, and *I*_SPTr_, *V*_post_, and *I*_QLED_ for all the pixels of the 10 × 10 images were acquired (fig. S12, A to C; each figure exemplifies the cases of pixels 1, 2, and 3, respectively). Then, the acquired *V*_post_ values were applied to the 10 × 10 passive matrix array of QLEDs to visualize the SoN on-device preprocessed image (fig. S12D). The SPTr produces the in-sensor preprocessed image with enhanced contrast, compared to noisy input images (Fig. 3Hii), but the in-sensor preprocessed image still contains background noise. However, the SoN on-device preprocessed image derived from the SPTr-QLED contains nearly zero background noise (Fig. 3Hiii, left). Furthermore, the QLEDs can visualize the preprocessed image (Fig. 3Hiii, right). These results demonstrate the photodetection, preprocessing, and visualization of UV patterns with reduced background noise by the SPTr-QLED.

### Recognition of SoN on-device preprocessed images by a deep neural network

**Fig. 4.**
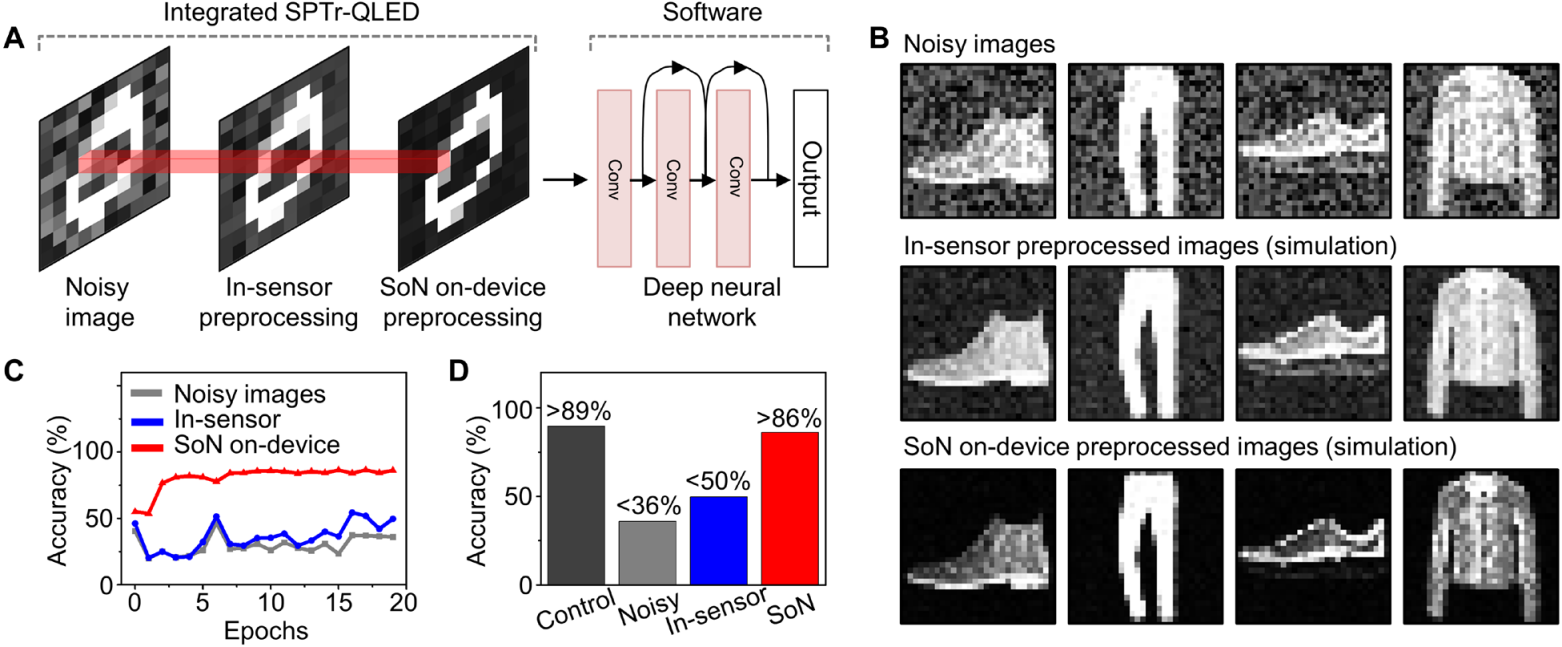
Image recognition demonstrations through simulations. (**A**) Schematic illustration showing the process for SoN on-device preprocessing by the SPTr-QLED and the recognition of the preprocessed image based on the deep neural network (i.e., ResNet50). (**B**) Noisy images prepared by modification of fashion images from the fashion MNIST test dataset, simulated images using an empirical parameter for in-sensor preprocessing, and simulated images using an empirical parameter for SoN on-device preprocessing. (**C**) Recognition rate of the noisy images, the in-sensor preprocessed images prepared by simulation, and the SoN on-device preprocessed images prepared by simulation over the number of epochs. (**D**) Recognition rate of noisy images, in-sensor preprocessed images prepared by simulation, and SoN on-device preprocessed images prepared by simulation. The recognition rate of raw images from the fashion MNIST test dataset is shown in the dark gray bar as a control.

For image recognition by a deep neural network, the quality of a target image that includes background noise is an important factor in determining the recognition accuracy. Because the neural network is typically trained with a standard training dataset of raw images without background noise, the background noise in target images can lead to false recognition. In particular, if the high noise level of raw UV images is considered, the reduction of the background noise is critical to the accurate image recognition. The SPTr-QLED can acquire a preprocessed image with reduced background noise without additional image-filtering steps during back-end processing and thus has potential for enhancing efficiency and accuracy of the image recognition by the deep neural network (i.e., ResNet50) (Fig. 4A) (*3*, *6*, *20*).

The noisy images were prepared through simulations that added a high–noise level background (σ ~ 0.5) to the raw images from the fashion MNIST test dataset (fig. S13) (*51*). Then, the in-sensor preprocessed images and SoN on-device preprocessed images were generated by the simulations using the empirical parameters obtained by fitting the calibration curves of the SPTr and SPTr-QLED (fig. S7, B and D), respectively. In comparison with the noisy images (Fig. 4B, top), the in-sensor preprocessing with the SPTr produced less noisy images, although the background noise still existed (Fig. 4B, middle). In the SoN on-device preprocessing case with the SPTr-QLED, however, even more vivid images with reduced background noise could be obtained (Fig. 4B, bottom). More details of the parametric fitting and the simulations for the image generation are provided in section S2.

The recognition accuracies of the three kinds of the prepared images by a deep neural network trained with the standard fashion MNIST training dataset were investigated (Fig. 4C). The training and inference processes were conducted by using software. After 20 training epochs, the recognition rate of the images prepared with the empirical parameter from the SPTr-QLED through simulation reached 86.2% (red bar in Fig. 4D), which was comparable to the recognition rate of the noiseless raw images (i.e., standard test dataset of fashion MNIST, 89.7%; black bar in Fig. 4D). However, the recognition rates of the noisy images were between 31 and 41%, with an average of 35.9% (gray bar in Fig. 4D and fig. S14A), indicating that the image recognition rate was seriously degraded by the background noise. Furthermore, the recognition rate of the in-sensor preprocessed images (SPTr case, prepared by simulation) was only 49.7% (blue bar in Fig. 4D), implying that the conventional SPD is insufficient for efficient recognition of noisy UV images, and thus, additional noise filtering is necessary to achieve the high recognition accuracy. The accuracy of each class of fashion items is shown in fig. S14 (B to D). In addition, even after the effect of the nonuniformity of the device array was considered, the recognition accuracy of the SoN on-device preprocessed images was not affected much (fig. S15 and section S3). The details of deep neural network training and recognition are provided in Materials and Methods.

## DISCUSSION

A device integrating SPTrs and QLEDs, inspired by the all-or-none potentiation of human synapses, was developed for photodetection, SoN on-device preprocessing, visualization, and recognition of noisy UV patterns. The SPTr-QLED could derive a preprocessed image from the frequent/strong optical signals by SoN on-device preprocessing, which could reduce background noise and consequently enhance the image recognition accuracy. Besides, the SPTr-QLED could visualize the high-contrast image obtained by SoN on-device preprocessing of acquired UV information. Meanwhile, for the scalable fabrication and commercialization of the SPTr-QLED, using Si complementary metal-oxide semiconductor fabrication technologies is important (*52*). However, there are still challenges relevant to the QLED fabrication including quantum dot patterning, material stability, and solvent orthogonality. Therefore, unconventional patterning techniques and various encapsulation strategies compatible with conventional Si fabrication processes need to be explored (*53*, *54*). In addition, as a future research topic, the development of an SPD with threshold switching characteristics would be helpful to reduce hardware complexity and improve efficiency of the SPTr-QLED.

## MATERIALS AND METHODS

### Fabrication of the integrated device of SPTr and QLEDs

The fabrication process of the SPTr-QLED is shown in fig. S3A. The fabrication of the SPTr began with the deposition and patterning of Al source/drain electrodes (30 nm) by thermal evaporation and wet etching. Then, a thin film of a-IGZO (30 nm) was deposited by sputtering through a shadow mask for the channel. The length and width of the a-IGZO channel were 5 and 910 μm, respectively. A pV3D3 thin film (30 nm) was deposited by initiated chemical vapor deposition for the dielectric layer (*55*). Ti/Au gate electrodes (5/20 nm) were deposited by thermal evaporation and patterned through a lift-off process. Parylene-C (1 μm) was deposited for encapsulation, and the electrical contact regions in the encapsulation layer were etched by reactive ion etching.

The fabrication of the QLED began with the deposition of Cr/Au (7/70 nm) anode contact lines on a glass substrate by thermal evaporation with a shadow mask. Indium tin oxide (ITO, 150 nm) anodes were sputtered on a glass substrate and patterned by a shadow mask. Poly(3,4-ethylenedioxythiophene):poly(styrene sulfonate) (Al 4083, Clevios) was spin-coated onto the plasma-treated ITO anodes and annealed at 180°C for 30 min. The following spin-coating and annealing steps were performed inside an Ar-filled glovebox to avoid unwanted material degradation induced by O_2_ and water molecules. Poly(9,9-dioctylfluorene-alt-*N*-(4-sec-butylphenyl)-diphenylamine (5 mg/ml in m-xylene) doped with 2,3,5,6-tetrafluoro-7,7,8,8-tetracyanoquinodimethane (1 mg/ml in m-xylene) was spin-coated and annealed at 180°C for 30 min. Quantum dots and ZnO nanoparticles were synthesized according to the previously reported method (*56*). Green CdSe/ZnS core/shell quantum dots were spin-coated and annealed at 180°C for 30 min. ZnO nanoparticles (6 mg/ml in 1-butanol) were spin-coated and annealed at 150°C for 30 min. Next, the sample was removed from the glovebox, and the deposition of Al cathodes (50 nm) by thermal evaporation with a shadow mask completed the fabrication of the QLED. Then, an encapsulation layer with a double-layer structure of Parylene-C and epoxy (SU8-2000.5, MicroChem, 1.5 μm) was deposited.

The SPTrs and QLEDs, individually fabricated on a glass substrate, were assembled by adhesive into a single device (fig. S3B). Each pixel of the SPTr and QLED was electrically connected to the external electronics (e.g., current-to-voltage converter) via anisotropic conductive films. The current-to-voltage converter consists of a transimpedance amplifier and an inverter, each of which was made of operational amplifiers (LMC662CN, Texas Instruments) and is powered by an Arduino Uno and a voltage regulator (LTC1983, Linear Technology). The *V*_post_ is converted from *I*_SPTr_ with an amplification ratio of *R*_amp_ (*V*_post_ = *I*_STPr_ × *R*_amp_; Fig. 2B). The overall system, including the integrated SPTr-QLED and external electronics, is shown in fig. S3C.

### Characterization of the SPTr and QLED

The electrical properties of the SPTr-QLED were measured using a multichannel data acquisition (DAQ) system (USB6289, National Instruments) and a parameter analyzer (B1500A, Agilent). The current-voltage curves and brightness of the QLEDs were measured using a source meter (Keithley 2436, Tektronix) and a spectrophotometer (CS-2000, Konica Minolta), respectively. A UV LED with a wavelength of 385 nm was used as the light source. For characterizing the wavelength-dependent photoresponse behavior of the SPTr-QLED, commercial LEDs with a wavelength of 625, 525, 475, 385, and 340 nm were used as light sources. The frequency-varied and intensity-varied optical inputs were programmed and generated using the Arduino Uno.

### Imaging and visualization demonstration using the integrated SPTr-QLED

The experimental setup for the 3 × 3 array demonstration of the integrated SPTr-QLED is shown in fig. S10. For the illumination of the UV patterns, a shadow mask with a shape of letter H or T was placed under the integrated SPTr-QLED, and the UV light from a commercial UV LED (*P*_UV_ = 4.36 μW cm^−2^) was irradiated through the shadow mask. In this array demonstration, each drain electrode of the SPTrs was connected to the individual current-to-voltage converter, the *I*_SPTr_ of each SPTr was converted into *V*_post_ by the current-to-voltage converter, and the *V*_post_ of nine pixels were measured using a multichannel DAQ system. Each *V*_post_ was also applied to the corresponding anode of the QLED. Because each QLED cathode line was connected to an individual current-to-voltage converter, the *I*_QLED_ of nine pixels could be also acquired as the multichannel DAQ system measured the voltage output converted from the *I*_QLED_ using the current-to-voltage converter. Photographs of the QLEDs were captured by an optical camera during the array demonstration.

To demonstrate the preprocessing of 10 noisy images of the pattern “0,” a unit device of the SPTr-QLED was used to scan the pixels in the 10 × 10 images one by one. The *V*_post_ was measured using the multichannel DAQ system. *I*_SPTr_ was obtained from measured *V*_post_ by dividing it with *R*_amp_. *I*_QLED_ was measured by using the parameter analyzer. For the visualization of the preprocessed image, the 10 × 10 passive matrix array of QLEDs was driven by *V*_post_ acquired from the aforementioned scanning method.

### Software-based image recognition by a trained deep neural network

The training and inference of the fashion MNIST dataset were conducted using software, e.g., Python with TensorFlow. For training the deep neural network (i.e., ResNet50 imported from Keras) at a software domain, Adam was used as an optimizer, sparse_categorical_crossentropy was used as a loss function, and 50,000 raw images from the fashion MNIST training dataset were used. The training images were resized into a shape of (32, 32, 1) to be used in ResNet50. Ten types of the noisy images (Fig. 4B, top, and fig. S13), the simulated in-sensor preprocessed images (Fig. 4B, middle), and the simulated SoN on-device preprocessed images (Fig. 4B, bottom) were prepared using simulations (section S2). The raw images in the fashion MNIST test dataset were prepared as a control. All these images were resized into a shape of (32, 32, 1) before evaluation. Then, the recognition rates of these images were evaluated at each training epoch (Fig. 4C). After 20 training epochs, the image recognition rates of the image datasets were measured to compare the improvements in image recognition (Fig. 4D) (*3*, *6*, *20*). The accuracies of the classes are also compared in fig. S14 (B to D).

## Supplementary Materials

### This PDF file includes:

Supplementary Text Figs. S1 to S15 References
