# Supplementary material for: Integration of synaptic phototransistors and quantum dot light-emitting diodes for visualization and recognition of UV patterns
Source: Sci Adv. 2022 Oct 12;8(41):eabq3101. doi: 10.1126/sciadv.abq3101 (PMC9555778; doi:10.1126/sciadv.abq3101)
Supplement: Supplementary file 1 — Supplementary Text Figs. S1 to S15 References [file sciadv.abq3101_sm.pdf]

Science Advances
AAAS

Supplementary Materials for

Integration of synaptic phototransistors and quantum dot light-emitting diodes for visualization and recognition of UV patterns

Hyojin Seung *et al.*

Corresponding author: Dae-Hyeong Kim, dkim98@snu.ac.kr; Taeghwan Hyeon, thyeon@snu.ac.kr;
Moon Kee Choi, mkchoi@unist.ac.kr

Sci. Adv. **8**, eabq3101 (2022)
DOI: 10.1126/sciadv.abq3101

The PDF file includes:

Supplementary Text
Figs. S1 to S15
Legend for movie S1
References

Other Supplementary Material for this manuscript includes the following:

Movie S1

Supplementary Text

Section S1. UV noise filtering in signal-or-none (SoN) on-device preprocessing

The SPTr-QLED could obtain the SoN on-device preprocessed image with significantly reduced background noise by highlighting the frequent/strong inputs as amplified signals and filtering out the infrequent/weak inputs. Therefore, the SoN on-device preprocessing is helpful for the specific UV imaging circumstances, where the meaningful signals feature strong and invariant intensity while the noises feature weak and varying intensity (fig. S2). It corresponds to the noisy UV imaging, where a lot of noise is generated by scattering and absorption of UV light at the object's surface (*33*). However, the SoN on-device preprocessing may not be appropriate for some UV imaging situations. It is because the infrequent and weak signals can be cut-off even if they include meaningful information, resulting in unwanted loss of information (fig. S11). In that case, the threshold voltage of the QLED can be tuned by using several strategies (*e.g.*, bandgap engineering of QDs) (*57*, *58*), and thus the small-intensity signal loss can be minimized by lowering the maximum level of the noise removed by the threshold cut of the QLED.

Section S2. Simulations for image generation based on empirically fitted parameters

In the image simulation, the fashion MNIST test dataset, consisting of 10 classes of images with a label (0 for T-shirt/top, 1 for trouser, 2 for pullover, 3 for dress, 4 for coat, 5 for sandal, 6 for shirt, 7 for sneaker, 8 for bag, and 9 for ankle boot), was used. To mimic the image acquisition under a noisy UV environment, the noise matrix of a 28 $\times$ 28 array, whose intensities ranged from 0 to σ (σ = 0.5), was generated randomly, and the noise matrix was added to each image in the fashion MNIST test dataset, whose intensities are normalized in a range from 0 to 1. An intensity

value of more than 1 was normalized to 1. The above process was repeated 10 times; thus, the 10 simulated noisy image datasets were generated (Fig. 4B, top and fig. S13).

To simulate the on-device preprocessing by SPTr and QLED, the dependence of I_{SPTr} on the irradiated UV intensity (P_{UV}) and the dependence of I_{QLED} on I_{SPTr} were analyzed by analytical fitting. According to the analytical fitting, I_{SPTr} was linearly dependent on the light intensity, *i.e.*, $I_{SPTr} = \alpha_{SPTr} \times P_{UV}$, where α_{SPTr} is 1.015 (fig. S8B), and the I_{QLED} was exponentially dependent on the I_{SPTr}, *i.e.*, $I_{QLED} = \alpha_{QLED} \times (\exp(I_{SPTr}/\tau_{QLED}) - 1)$, where α_{QLED} is 0.01476 and τ_{QLED} is 0.2356 (fig. S8D). Then, the in-sensor preprocessed image was simulated by adding the I_{SPTr} induced by the illumination of each image in the 10 noisy image datasets (Fig. 4B, middle). The I_{SPTr} induced by image illumination was calculated based on the linear relationship between I_{SPTr} and P_{UV}. In addition, the SoN on-device preprocessed image was simulated from the in-sensor preprocessed image based on the exponential relationship between I_{QLED} and I_{SPTr} (Fig. 4B, bottom).

Section S3. Effect of device non-uniformity on the image recognition accuracy

In order to consider effect of the device non-uniformity in the array, we simulated the in-sensor preprocessed images and the SoN on-device preprocessed images using the fitted parameters (*e.g.*, α_{SPTr}, α_{QLED}, and τ_{QLED}) with a normal distribution (figs. S15A and S15B). The average of α_{SPTr}, α_{QLED}, and τ_{QLED} was set to 1.015, 0.01476, and 0.2356, respectively, and the standard deviation of the normal distribution was set to 1%, 5%, or 10%. The recognition accuracies of the preprocessed images were investigated using a deep neural network trained with a standard fashion MNIST dataset. The preprocessed images (figs. S15A and S15B) showed the similar recognition rates to those of the preprocessed images obtained without considering the

device's non-uniformity (Fig. 4B, middle and bottom), indicating that the image recognition can be improved by the SPTr-QLED regardless of the device-to-device variations (figs. 15C and 15D).

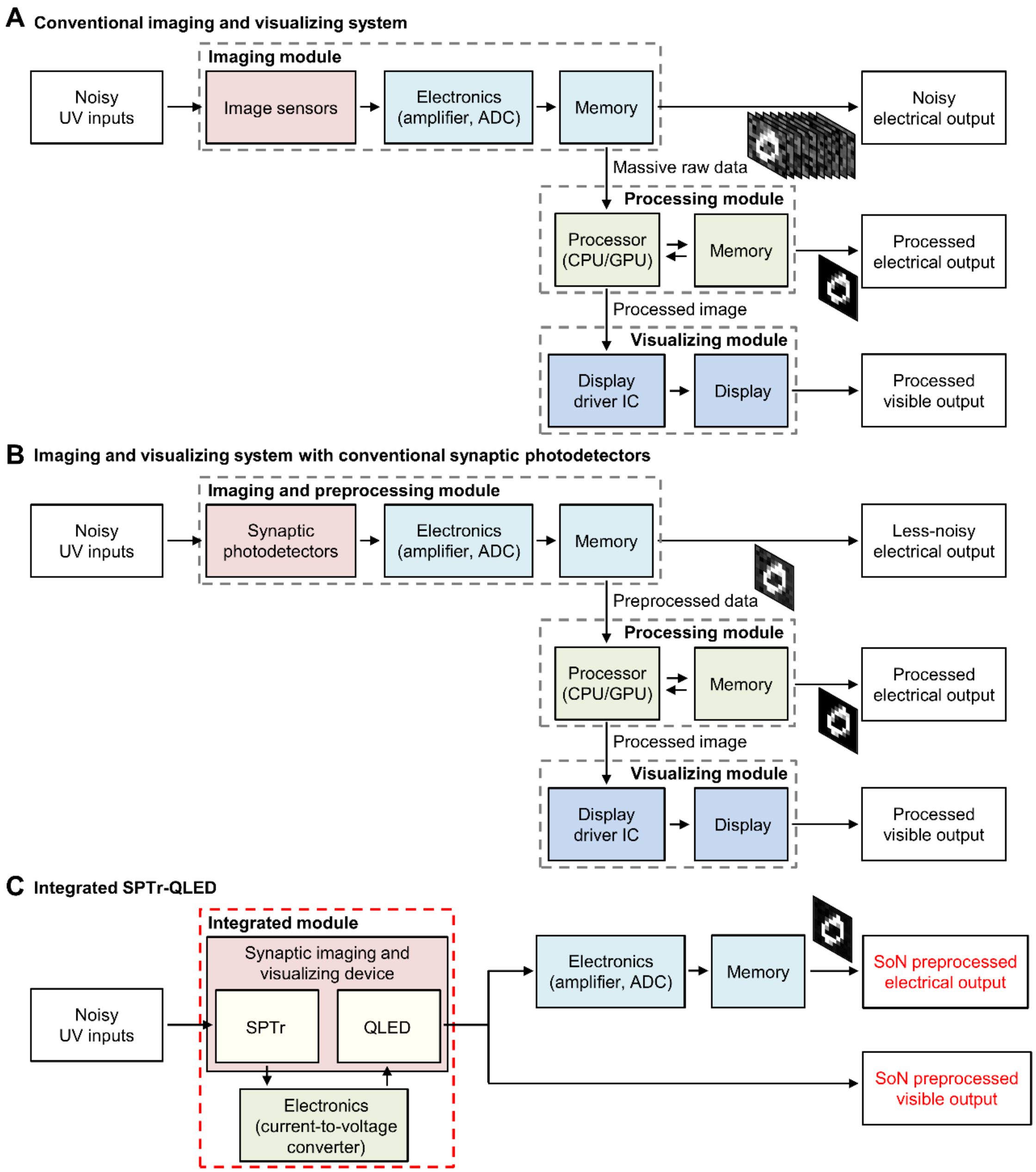

Fig. S1. Schematic diagrams of the image acquisition, data processing, and visualization systems. (**A** to **C**) UV image acquisition, processing, and visualization by the conventional imaging and visualizing system (A), the imaging and visualization system with conventional synaptic photodetectors (B), the system with the integrated SPTr-QLED (C).

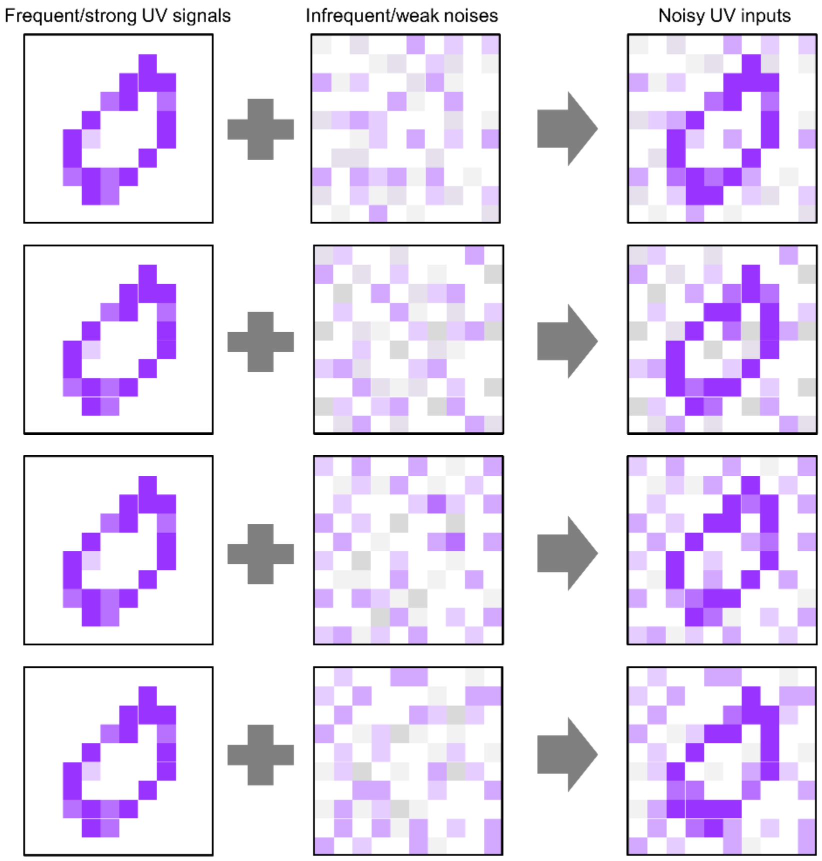

Fig. S2. Noisy UV patterns. Noisy UV inputs consisting of frequent/strong UV signals and infrequent/weak background noises.

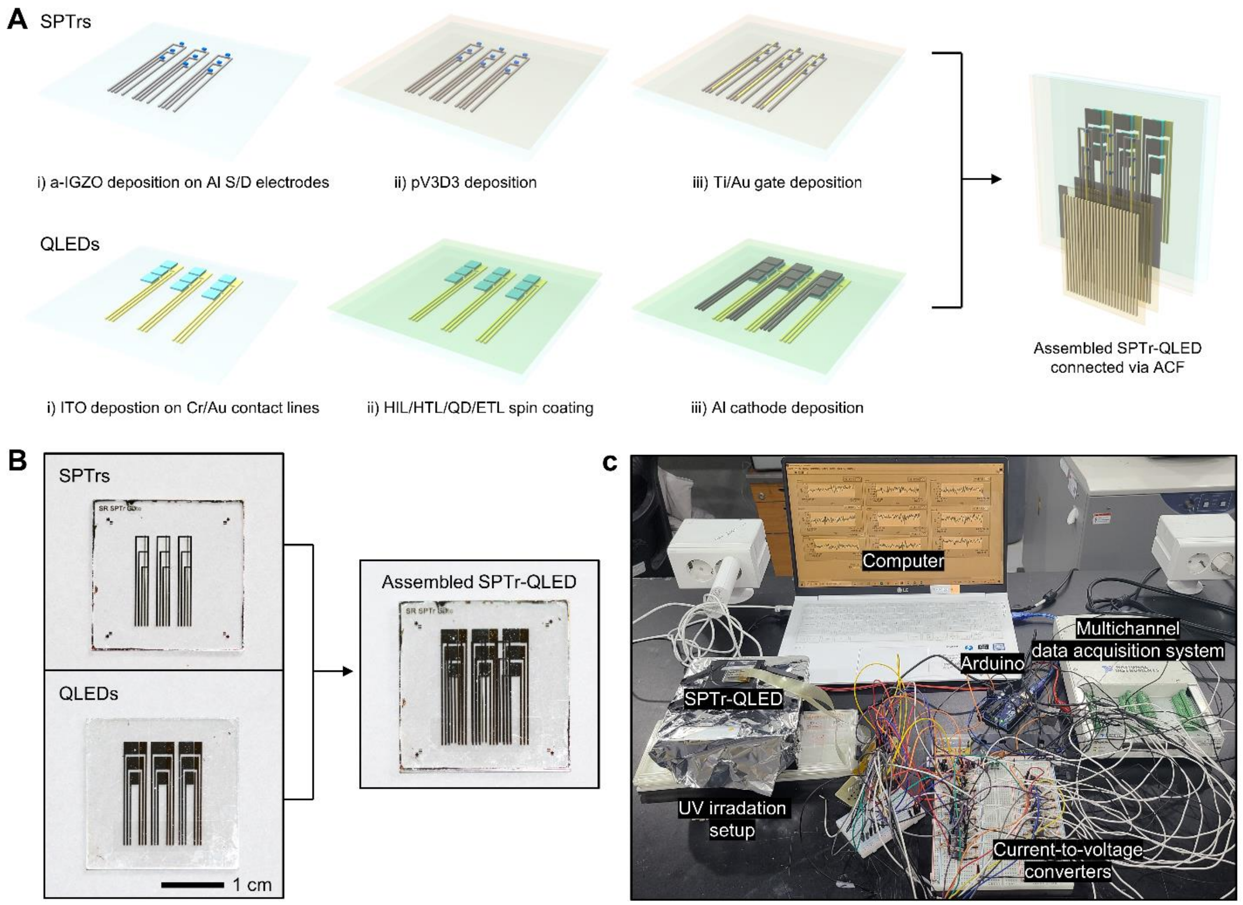

Fig. S3. Integrated device of synaptic phototransistor and quantum dot light-emitting diode. (**A**) Schematic illustration of the fabrication process of the integrated SPTr-QLED. (**B**) Photographs of SPTrs (left top), QLEDs (left bottom), and their assembled device (right). (**C**) Photograph of the overall system, including the integrated SPTr-QLED connected to the external electronics and the UV irradiation setup.

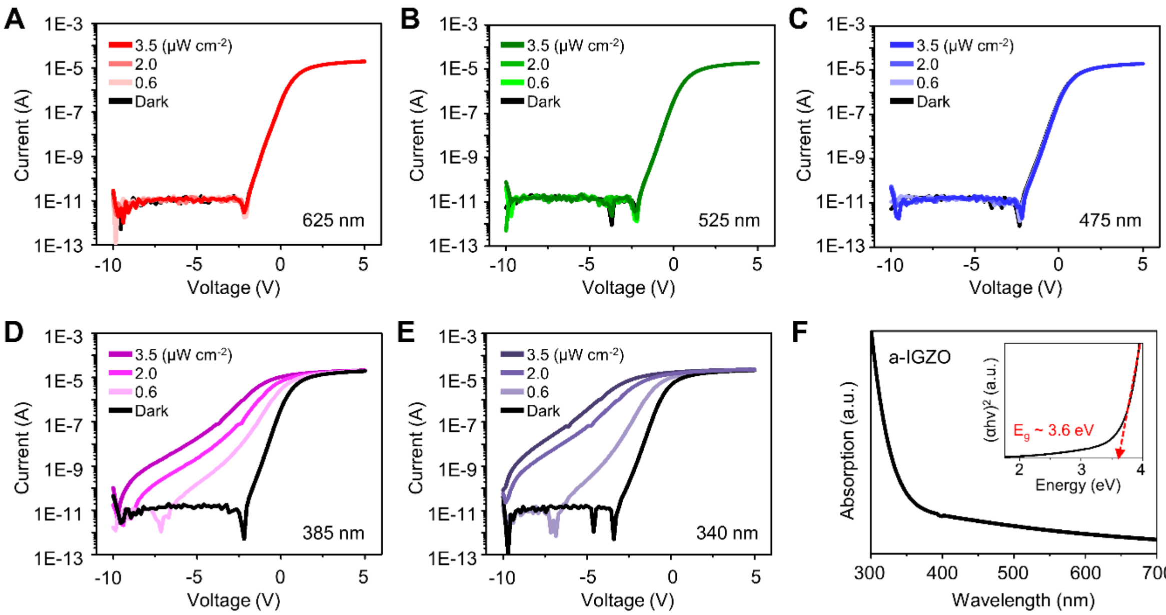

Fig. S4. Wavelength-dependent photoresponse of the synaptic phototransistor. (**A** to **E**) Transfer curves of the synaptic phototransistor upon the irradiation of different wavelength lights, *i.e.*, 625 nm (A), 525 nm (B), 475 nm (C), 385 nm (D), and 340 nm (E). For each wavelength, light inputs at different intensities (*i.e.*, dark, 0.6 μW cm^{-2}, 2.0 μW cm^{-2}, and 3.5 μW cm^{-2}) were used to measure the intensity-dependent photoresponse of the synaptic phototransistor. (**F**) UV-visible absorption spectra of a-IGZO. The inset shows a Tauc plot, indicating the bandgap of a-IGZO (~ 3.6 eV).

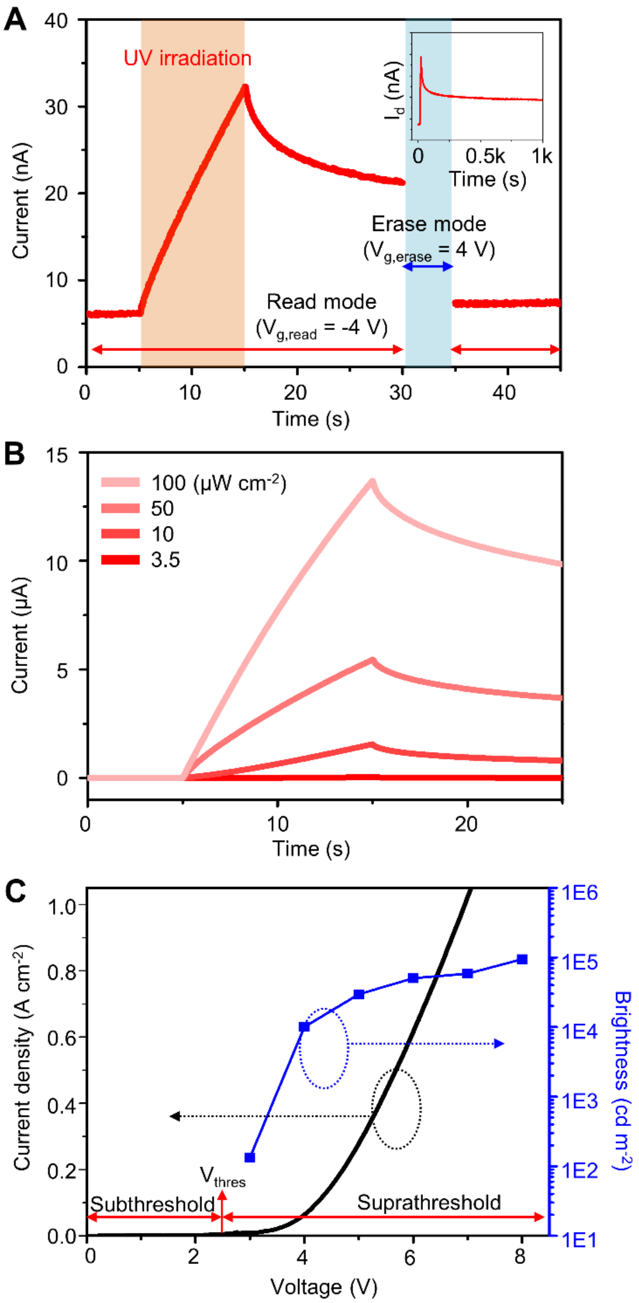

Fig. S5. Device characteristics of the synaptic phototransistor and quantum dot light-emitting diode. (**A**) UV-stimulated (intensity = 2.75 µW cm^{-2}, exposure time = 10 s) photocurrent generation and decay of the SPTr under a negative gate bias (V_d = 1 V, $V_{g,read}$ = −4 V, read mode). The photocurrent is recovered to its original state by applying a positive gate bias ($V_{g,erase}$ = 4 V, erase mode). The inset shows the slow decay characteristics after the removal of the UV illumination. (**B**) Photocurrent measurement of the SPTr in response to the UV irradiations with various light intensities (*e.g.*, 3.5 µW cm^{-2}, 10 µW cm^{-2}, 50 µW cm^{-2}, and 100 µW cm^{-2}). (**C**) Current-density–voltage–luminance (*J*–*V*–*L*) characteristics of the QLED, marked with V_{thres} (~ 2.5 V).

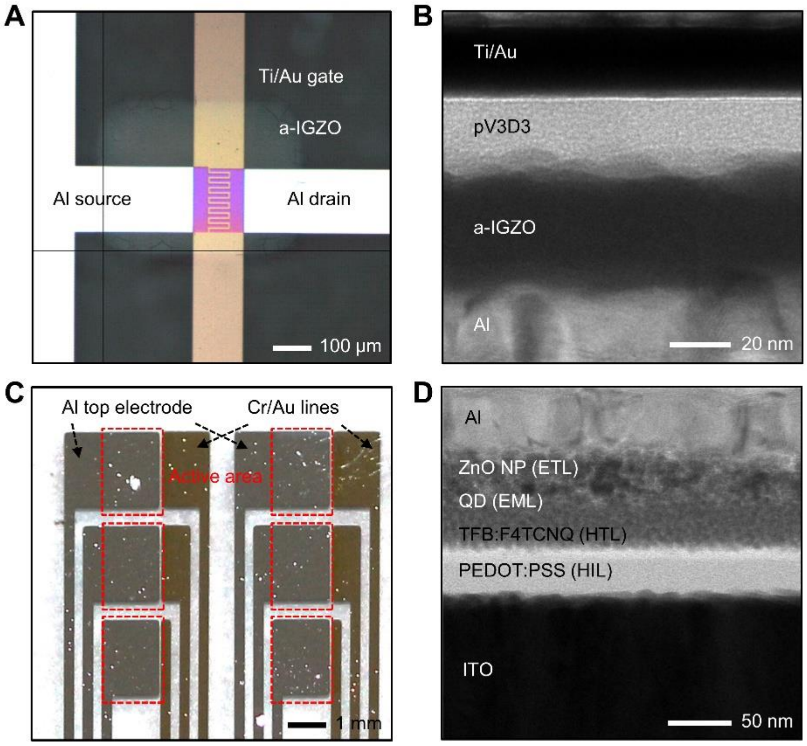

Fig. S6. Images of the synaptic phototransistor and quantum dot light-emitting diode. (**A** and **B**) The optical microscopy image (A) and cross-sectional TEM image (B) showing the lateral and vertical structures of the SPTr, respectively. (**C** and **D**) Photograph (C) and cross-sectional TEM image (D) showing the lateral and vertical structures of the QLED, respectively.

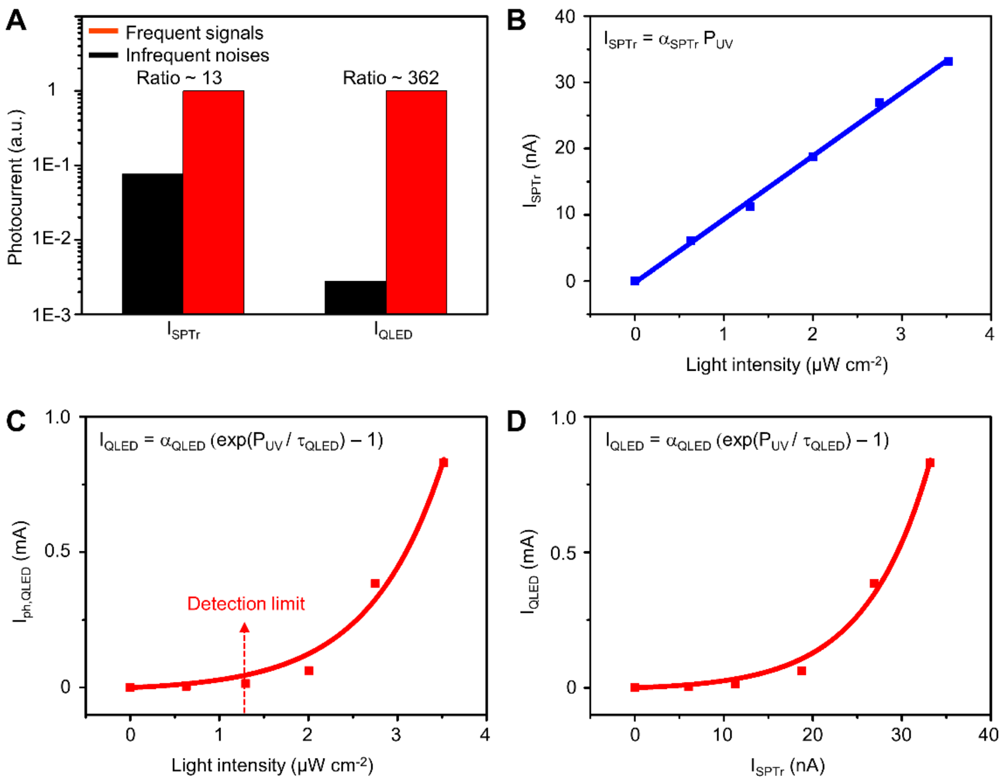

Fig. S7. Characteristics of on-device preprocessing. (**A**) The normalized photocurrents (I_{SPTr} and I_{QLED}) generated by frequent signals (*i.e.*, 20 UV pulses with a duration of 0.5 s and a frequency of 1.0 Hz) and infrequent noises (*i.e.*, two UV pulses with a duration of 0.5 s and a frequency of 0.1 Hz). Each photocurrent is normalized based on the photocurrents generated by the frequent signals. The ratios for the I_{SPTr} and I_{QLED} are approximately 13 and 362, respectively. (**B** and **C**) I_{SPTr} (B) and I_{QLED} (C) depending on the light intensity. (**D**) The exponential dependence of I_{SPTr} and I_{QLED}, each of which is the result of the conventional in-sensor preprocessing and SoN on-device preprocessing, respectively.

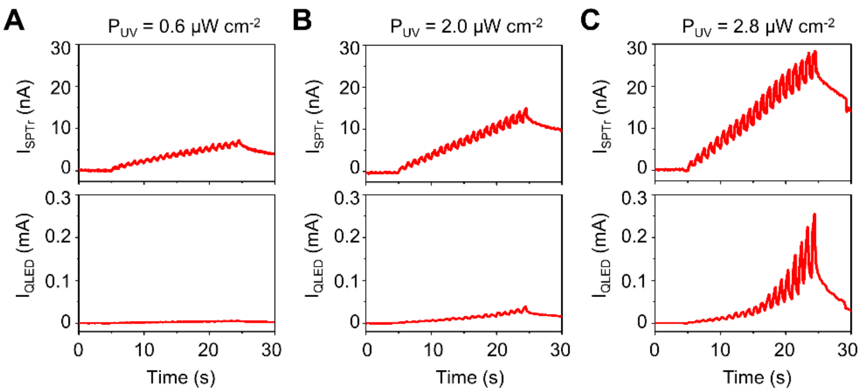

Fig. S8. Current response of the synaptic phototransistor and the quantum dot light-emitting diode upon the irradiation of the pulsed UV light. (**A** to **C**) I_{SPTr} and I_{QLED} induced by the frequent UV light inputs (*i.e.*, 20 UV pulses with a duration of 0.5 s and a frequency of 1.0 Hz) with different intensities (*i.e.*, 0.6 μW cm^{-2} (A), 2.0 μW cm^{-2} (B), and 2.8 μW cm^{-2} (C)).

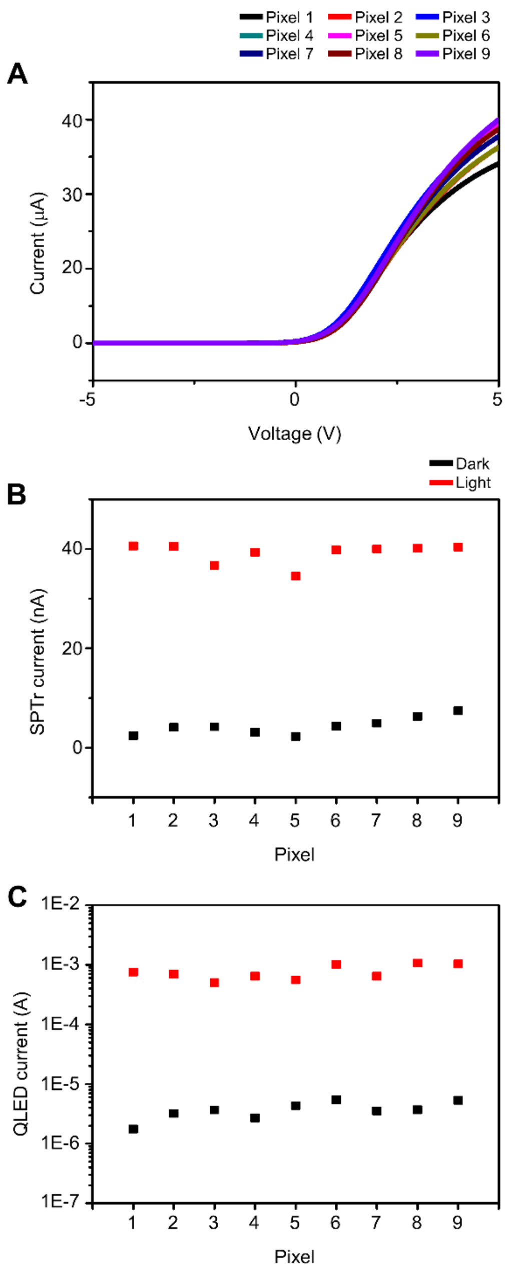

Fig. S9. Uniformity of the integrated device array. (**A**) Transfer curves of nine SPTrs in the integrated device array. (**B** and **C**) Dark current and photocurrent of the SPTrs (B) and the QLEDs (C) with the irradiation of 20 frequent pulsed UV light inputs (P_{UV} = 4.36 μW cm^{-2}).

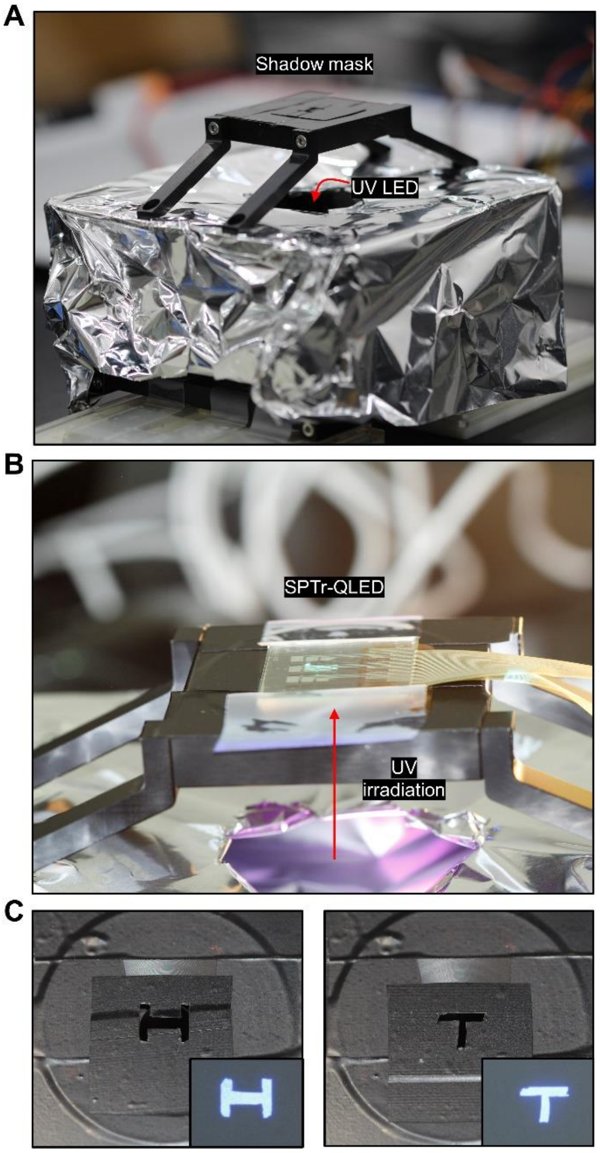

Fig. S10. The experimental setup for array demonstration. (**A**) Photograph of the UV irradiation setup. (**B**) Photograph of the SPTr-QLED irradiated with UV through the shadow mask. (**C**) Photographs of shadow mask of "H" shape (left) and shadow mask of "T" shape (right). Each inset shows the "H"- and "T"-shaped UV inputs.

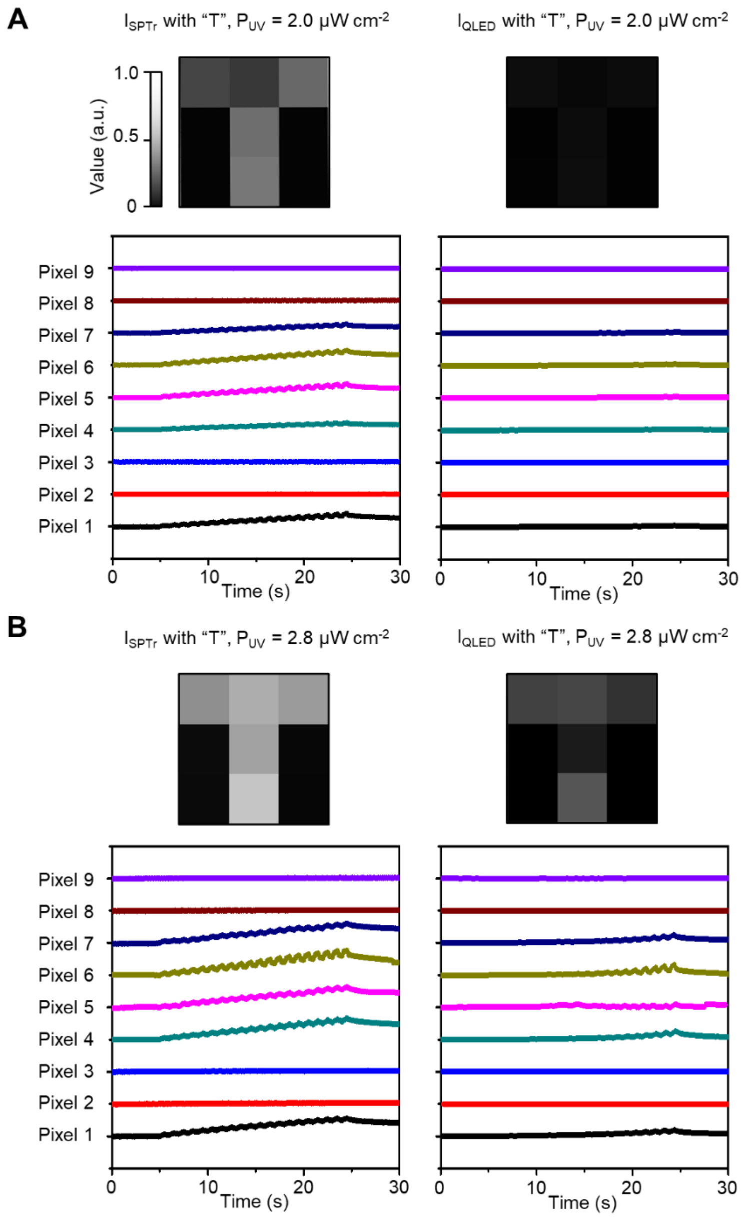

Fig. S11. Array demonstration of the SPTr-QLED. (**A** and **B**) I_{SPTr} and I_{QLED} of nine pixels in the 3 × 3 array during the frequent irradiation of pulsed UV light inputs with the pattern shape of "T" for two different UV light intensities (2.0 μW cm^{-2} (A) and 2.8 μW cm^{-2} (B), respsectively).

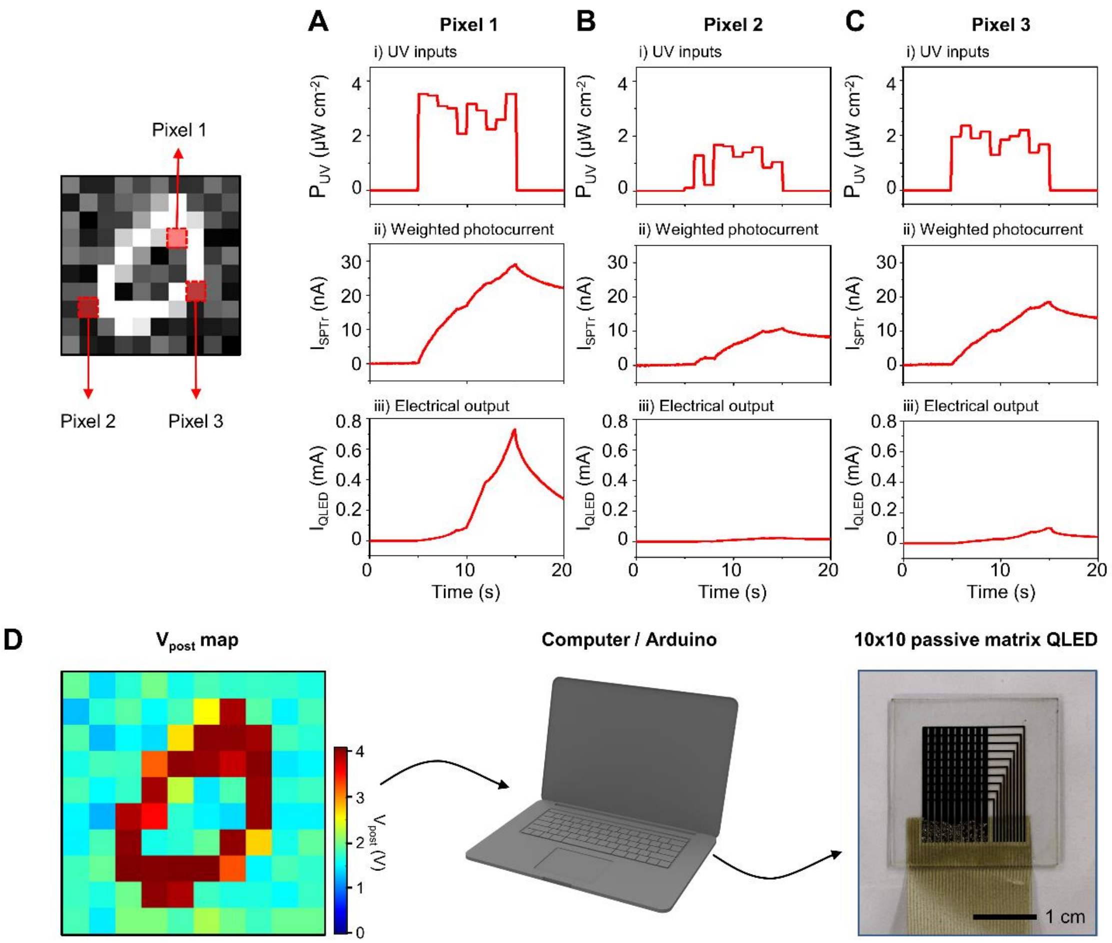

Fig. S12. Image acquisition and visualization demonstration of the sequentially irradiated noisy UV inputs. (**A** to **C**) The programmed UV inputs (i), each of which corresponds to the different pixels in the noisy images, I_{SPTr} (ii), and I_{QLED} (iii) of three representative pixels marked in the left image. Each pixel in the array is scanned one by one for the programmed UV irradiation. (**D**) Schematic illustration showing the visualization of 10 × 10 QLED passive matrix array based on the acquired V_{post} map. The V_{post} values were applied to the 10 × 10 QLED array to visualize the SoN on-device preprocessed image.

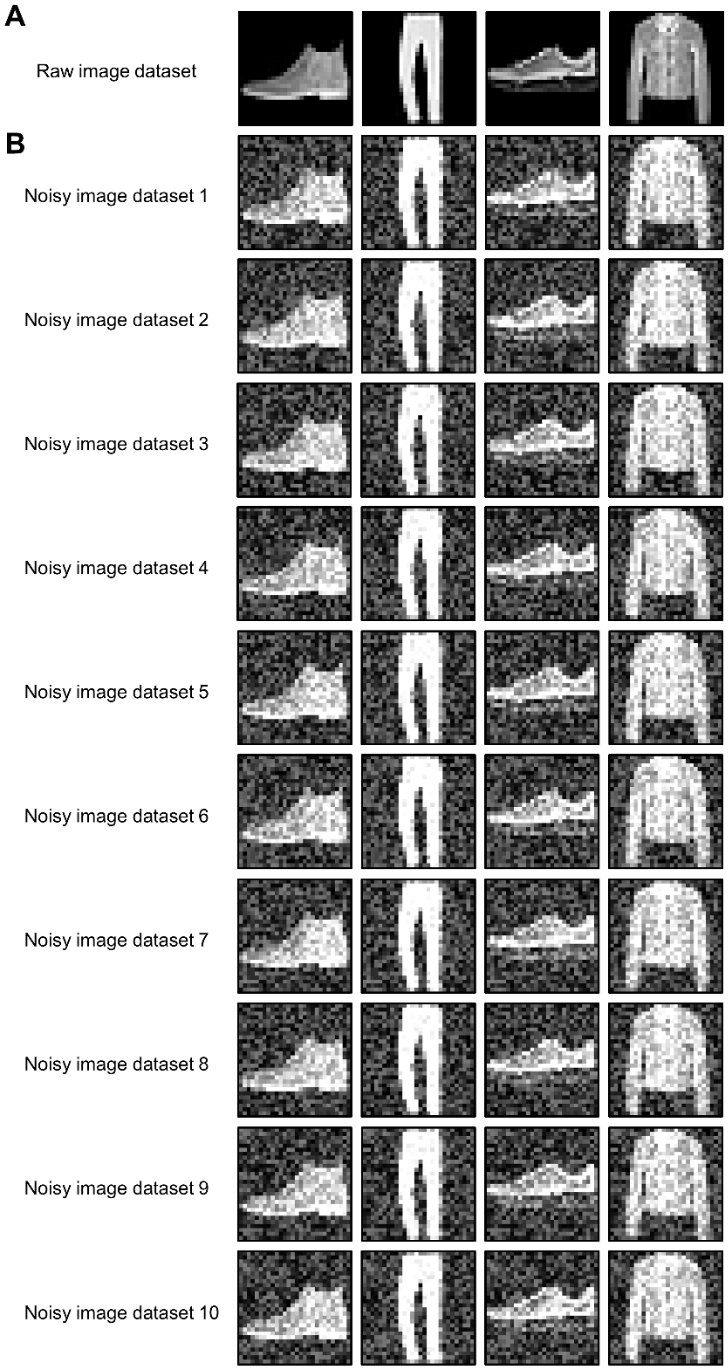

Fig. S13. Simulated noisy images. (**A**) Raw images from the fashion MNIST test dataset. (**B**) Noisy image sets with a high noise level ($\sigma = 0.5$). The randomly generated background noise is added to the raw images.

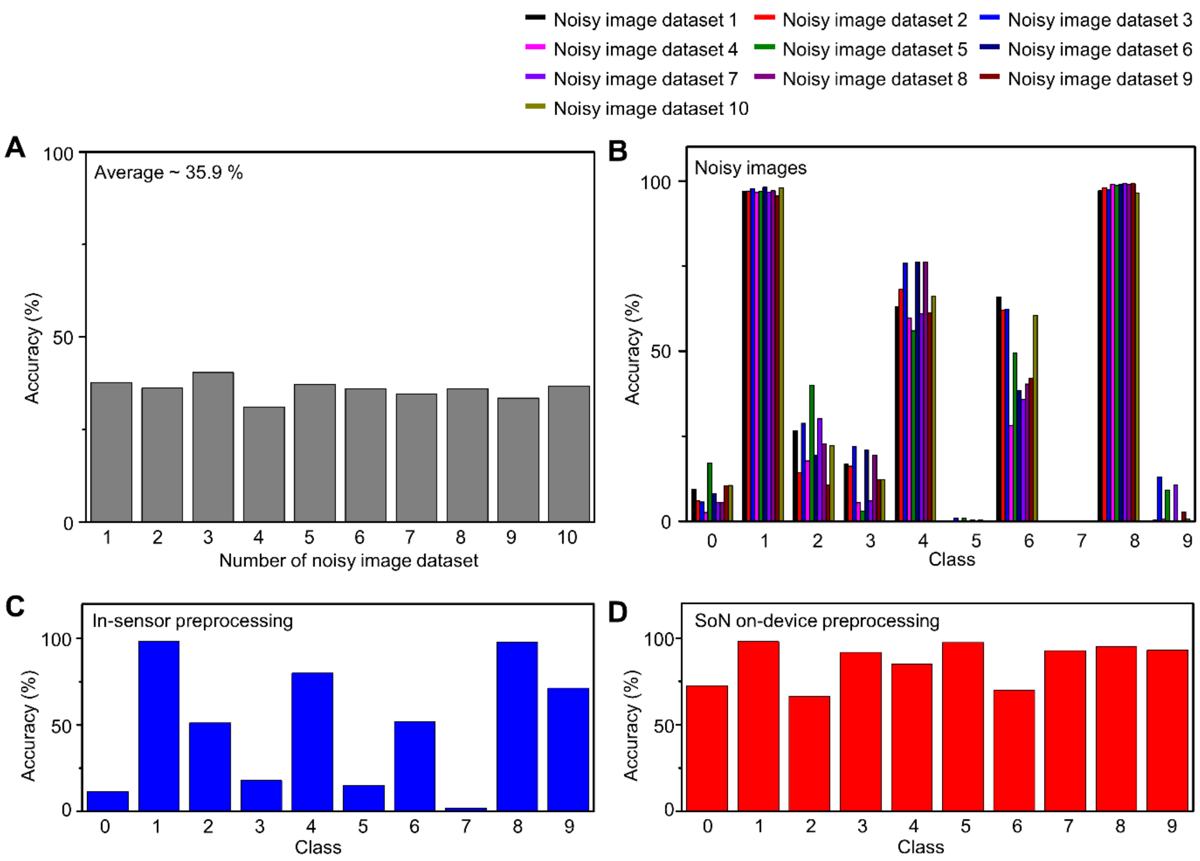

Fig. S14. Recognition rate. (**A**) Recognition rate of each noisy image dataset. The ResNet50 model trained with the fashion MNIST training dataset is used for the evaluation. (**B** to **D**) The recognition rate for each class of noisy images in 10 different datasets (B), the simulated in-sensor preprocessed images (C), and the simulated SoN on-device preprocessed images (D).

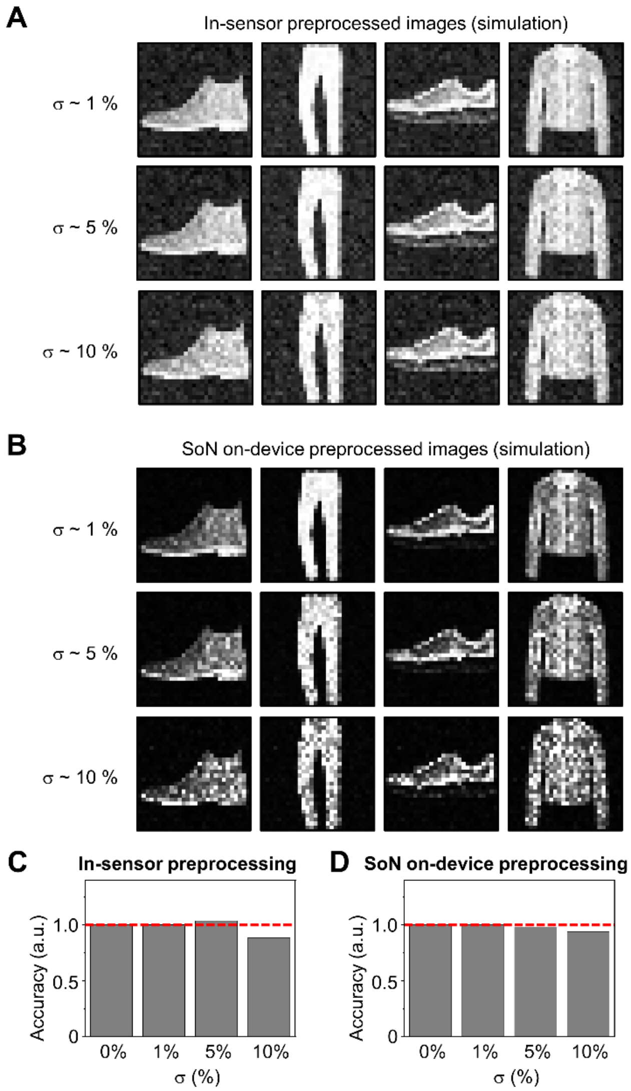

Fig. S15. Image recognition accuracy change after considering device-to-device variations. (**A, B**) The simulated images for the in-sensor preprocessing (A) and the SoN on-device preprocessing obtained using the fitted parameters with a normal distribution. The standard deviation for each image simulation was set to 1% (top), 5% (middle), and 10% (bottom), respectively. (**C, D**) Recognition rates of the in-sensor preprocessed images (C) and the SoN on-device preprocessed images (D), obtained using the fitted parameters with a normal distribution. The recognition rates for the 0% standard deviation were obtained using the fitted parameters without considering the device-to-device variation. The preprocessed images showed the similar recognition rates even after considering the device-to-device variations with those of the preprocessed images obtained without considering the device non-uniformity.

Movie S1.

The visible output of QLEDs during the frequent irradiation of UV pattern “T” and the infrequent irradiation of UV pattern “H”.

REFERENCES AND NOTES

1. S. Lany, A. Zunger, Anion vacancies as a source of persistent photoconductivity in II-VI and chalcopyrite semiconductors. *Phys. Rev. B* **72**, 035215 (2005).

2. M. Lee, W. Lee, S. Choi, J. W. Jo, J. Kim, S. K. Park, Y.-H. Kim, Brain-inspired photonic neuromorphic devices using photodynamic amorphous oxide semiconductors and their persistent photoconductivity. *Adv. Mater.* **29**, 1700951 (2017).

3. F. Zhou, Z. Zhou, J. Chen, T. H. Choy, J. Wang, N. Zhang, Z. Lin, S. Yu, J. Kang, H.-S. P. Wong, Y. Chai, Optoelectronic resistive random access memory for neuromorphic vision sensors. *Nat. Nanotechnol.* **14**, 776–782 (2019).

4. H. Jang, C. Liu, H. Hinton, M.-H. Lee, H. Kim, M. Seol, H.-J. Shin, S. Park, D. Ham, An atomically thin optoelectronic machine vision processor. *Adv. Mater.* **32**, 2002431 (2020).

5. L. Mennel, J. Symonowicz, S. Wachter, D. K. Polyushkin, A. J. Molina-Mendoza, T. Mueller, Ultrafast machine vision with 2D material neural network image sensors. *Nature* **579**, 62–66 (2020).

6. Z. Zhang, S. Wang, C. Liu, R. Xie, W. Hu, P. Zhou, All-in-one two-dimensional retinomorphic hardware device for motion detection and recognition. *Nat. Nanotechnol.* **17**, 27–32 (2021).

7. D. Jayachandran, A. Oberoi, A. Sebastian, T. H. Choudhury, B. Shankar, J. M. Redwing, S. Das, A low-power biomimetic collision detector based on an in-memory molybdenum disulfide photodetector. *Nat. Electron.* **3**, 646–655 (2020).

8. T. Ohno, T. Hasegawa, T. Tsuruoka, K. Terabe, J. K. Gimzewski, M. Aono, Short-term plasticity and long-term potentiation mimicked in single inorganic synapses. *Nat. Mater.* **10**, 591–595 (2011).

9. C. Choi, J. Leem, M. S. Kim, A. Taqieddin, C. Cho, K. W. Cho, G. J. Lee, H. Seung, H. J. Bae, Y. M. Song, T. Hyeon, N. R. Aluru, S. W. Nam, D.-H. Kim, Curved neuromorphic

image sensor array using a MoS_2-organic heterostructure inspired by the human visual recognition system. *Nat. Commun.* **11**, 5934 (2020).

10. Q.-B. Zhu, B. Li, D.-D. Yang, C. Liu, S. Feng, M.-L. Chen, Y. Sun, Y.-N. Tian, X. Su, X.-M. Wang, S. Qiu, Q.-W. Li, X.-M. Li, H.-B. Zeng, H.-M. Cheng, D.-M. Sun, A flexible ultrasensitive optoelectronic sensor array for neuromorphic vision systems. *Nat. Commun.* **12**, 1798 (2021).

11. Y. Chen, H. Wang, Y. Yao, Y. Wang, C. Ma, P. Samorì, Synaptic plasticity powering long-afterglow organic light-emitting transistors. *Adv. Mater.* **33**, 2103369 (2021).

12. K. Lee, H. Han, Y. Kim, J. Park, S. Jang, H. Lee, S. W. Lee, H. Y. Kim, Y. Kim, T. Kim, D. Kim, G. Wang, C. Park, Retina-inspired structurally tunable synaptic perovskite nanocones. *Adv. Funct. Mater.* **31**, 2105596 (2021).

13. H.-L. Park, H. Kim, D. Lim, H. Zhou, Y.-H. Kim, Y. Lee, S. Park, T.-W. Lee, Retina-inspired carbon nitride-based photonic synapses for selective detection of UV light. *Adv. Mater.* **32**, e1906899 (2020).

14. S. Kim, D. G. Roe, Y. Y. Choi, H. Woo, J. Park, J. I. Lee, Y. Choi, S. B. Jo, M. S. Kang, Y. J. Song, S. Jeong, J. H. Cho, Artificial stimulus-response system capable of conscious response. *Sci. Adv.* **7**, eabe3996 (2021).

15. D. Ham, H. Park, S. Hwang, K. Kim, Neuromorphic electronics based on copying and pasting the brain. *Nat. Electron.* **4**, 635–644 (2021).

16. Q. Xia, J. J. Yang, Memristive crossbar arrays for brain-inspired computing. *Nat. Mater.* **18**, 309–323 (2019).

17. H. Yeon, P. Lin, C. Choi, S. H. Tan, Y. Park, D. Lee, J. Lee, F. Xu, B. Gao, H. Wu, H. Qian, Y. Nie, S. Kim, J. Kim, Alloying conducting channels for reliable neuromorphic computing. *Nat. Nanotechnol.* **15**, 574–579 (2020).

18. M. A. Zidan, J. P. Strachan, W. D. Lu, The future of electronics based on memristive systems. *Nat. Electron.* **1**, 22–29 (2018).

19. F. Zhou, Y. Chai, Near-sensor and in-sensor computing. *Nat. Electron* **3**, 664–671 (2020).

20. F. Liao, Z. Zhou, B. J. Kim, J. Chen, J. Wang, T. Wan, Y. Zhou, A. T. Hoang, C. Wang, J. Kang, J.-H. Ahn, Y. Chai, Bioinspired in-sensor visual adaptation for accurate perception. *Nat. Electron.* **5**, 84–91 (2022).

21. S. H. Kim, G. W. Baek, J. Yoon, S. Seo, J. Park, D. Hahm, J. H. Chang, D. Seong, H. Seo, S. Oh, K. Kim, H. Jung, Y. Oh, H. W. Baac, B. Alimkhanuly, W. K. Bae, S. Lee, M. Lee, J. Kwak, J.-H. Park, D. Son, A bioinspired stretchable sensory-neuromorphic system. *Adv. Mater.* **33**, 2104690 (2021).

22. S. M. Kwon, J. Y. Kwak, S. Song, J. Kim, C. Jo, S. S. Cho, S.-J. Nam, J. Kim, G.-S. Park, Y.-H. Kim, S. K. Park, Large-area pixelized optoelectronic neuromorphic devices with multispectral light-modulated bidirectional synaptic circuits. *Adv. Mater.* **33**, 2105017 (2021).

23. S. Seo, S.-H. Jo, S. Kim, J. Shim, S. Oh, J.-H. Kim, K. Heo, J.-W. Choi, C. Choi, S. Oh, D. Kuzum, H.-S. P. Wong, J.-H. Park, Artificial optic-neural synapse for colored and color-mixed pattern recognition. *Nat. Commun.* **9**, 5106 (2018).

24. L. Gu, S. Poddar, Y. Lin, Z. Long, D. Zhang, Q. Zhang, L. Shu, X. Qiu, M. Kam, A. Javey, Z. Fan, A biomimetic eye with a hemispherical perovskite nanowire array retina. *Nature* **581**, 278–282 (2020).

25. Z. Rao, Y. Lu, Z. Li, K. Sim, Z. Ma, J. Xiao, C. Yu, Curvy, shape-adaptive imagers based on printed optoelectronic pixels with a kirigami design. *Nat. Electron.* **4**, 513–521 (2021).

26. M. S. Kim, G. J. Lee, C. Choi, M. S. Kim, M. Lee, S. Liu, K. W. Cho, H. M. Kim, H. Cho, M. K. Choi, N. Lu, Y. M. Song, D.-H. Kim, An aquatic-vision-inspired camera based on a monocentric lens and a silicon nanorod photodiode array. *Nat. Electron.* **3**, 546–553 (2020).

27. W. Lee, Y. Liu, Y. Lee, B. K. Sharma, S. M. Shinde, S. D. Kim, K. Nan, Z. Yan, M. Han, Y. Huang, Y. Zhang, J.-H. Ahn, J. A. Rogers, Two-dimensional materials in functional three-dimensional architectures with applications in photodetection and imaging. *Nat. Commun.* **9**, 1417 (2018).

28. Y. M. Song, Y. Xie, V. Malyarchuk, J. Xiao, I. Jung, K.-J. Choi, Z. Liu, H. Park, C. Lu, R.-H. Kim, R. Li, K. B. Crozier, Y. Huang, J. A. Rogers, Digital cameras with designs inspired by the arthropod eye. *Nature* **497**, 95–99 (2013).

29. C. Choi, M. K. Choi, S. Liu, M. S. Kim, O. K. Park, C. Im, J. Kim, X. Qin, G. J. Lee, K. W. Cho, M. Kim, E. Joh, J. Lee, D. Son, S.-H. Kwon, N. L. Jeon, Y. M. Song, N. Lu, D.-H. Kim, Human eye-inspired soft optoelectronic device using high-density MoS_2-graphene curved image sensor array. *Nat. Commun.* **8**, 1664 (2017).

30. R. E. Barsley, M. H. West, J. A. Fair, Forensic photography. Ultraviolet imaging of wounds on skin. *Am. J. Forensic Med. Pathol.* **11**, 300–308 (1990).

31. T. Mori, M. Burton, The SO_2 camera: A simple, fast and cheap method for ground-based imaging of SO_2 in volcanic plumes. *Geophys. Res. Lett.* **33**, L2480 (2006).

32. K. Kojima, K. Shido, G. Tamiya, K. Yamasaki, K. Kinoshita, S. Aiba, Facial UV photo imaging for skin pigmentation assessment using conditional generative adversarial networks. *Sci. Rep.* **11**, 1213 (2021).

33. T. Mori, T. Mori, K. Kazahaya, M. Ohwada, J. Hirabayashi, S. Yoshikawa, Effect of UV scattering on SO_2 emission rate measurements. *Geophys. Res. Lett.* **33**, L17315 (2006).

34. H. Yoo, I. S. Lee, S. Jung, S. M. Rho, B. H. Kang, H. J. Kim, A review of phototransistors using metal oxide semiconductors: Research progress and future directions. *Adv. Mater.* **33**, 2006091 (2021).

35. S. Hong, H. Cho, B. H. Kang, K. Park, D. Akinwande, H. J. Kim, S. Kim, Neuromorphic active pixel image sensor array for visual memory. *ACS Nano* **15**, 15362–15370 (2021).

36. J. Sun, S. Oh, Y. Choi, S. Seo, M. J. Oh, M. Lee, W. B. Lee, P. J. Yoo, J. H. Cho, J.-H. Park, Optoelectronic synapse based on IGZO-alkylated graphene oxide hybrid structure. *Adv. Funct. Mater.* **28**, 1804397 (2018).

37. H. Yabuta, M. Sano, K. Abe, T. Aiba, T. Den, H. Kumomi, K. Nomura, T. Kamiya, H. Hosono, High-mobility thin-film transistor with amorphous $InGaZnO_4$ channel fabricated by room temperature rf-magnetron sputtering. *Appl. Phys. Lett.* **89**, 112123 (2006).

38. K. Nomura, H. Ohta, A. Takagi, T. Kamiya, M. Hirano, H. Hosono, Room-temperature fabrication of transparent flexible thin-film transistors using amorphous oxide semiconductors. *Nature* **432**, 488–492 (2004).

39. M. S. Kim, M. S. Kim, G. J. Lee, S.-H. Sunwoo, S. Chang, Y. M. Song, D.-H. Kim, Bio-inspired artificial vision and neuromorphic image processing devices. *Adv. Mater. Technol.* 2100144 (2021).

40. G. J. Lee, C. Choi, D.-H. Kim, Y. M. Song, Bioinspired artificial eyes: Optic components, digital cameras, and visual prostheses. *Adv. Funct. Mater.* **28**, 1705202 (2018).

41. J. Park, H. Seung, D. C. Kim, M. S. Kim, D.-H. Kim, Unconventional image-sensing and light-emitting devices for extended reality. *Adv. Funct. Mater.* **31**, 2009281 (2021).

42. K. He, Y. Liu, M. Wang, G. Chen, Y. Jiang, J. Yu, C. Wan, D. Qi, M. Xiao, W. Ru Leow, H. Yang, M. Antonietti, X. Chen, An artificial somatic reflex arc. *Adv. Mater.* **32**, e1905399 (2020).

43. A. E. Pereda, Electrical synapses and their functional interactions with chemical synapses. *Nat. Rev. Neurosci.* **15**, 250–263 (2014).

44. A. Citri, R. C. Malenka, Synaptic plasticity: Multiple forms, functions, and mechanisms. *Neuropsychopharmacology* **33**, 18–41 (2008).

45. J. Abbott, T. Ye, K. Krenek, R. S. Gertner, S. Ban, Y. Kim, L. Qin, W. Wu, H. Park, D. Ham, A nanoelectrode array for obtaining intracellular recordings from thousands of connected neurons. *Nat. Biomed. Eng.* **4**, 232–241 (2020).

46. K. Lucas, The "all or none" contraction of the amphibian skeletal muscle fibre. *J. Physiol.* **38**, 113–133 (1909).

47. H. Lee, B. G. Jeong, W. K. Bae, D. C. Lee, J. Lim, Surface state-induced barrierless carrier injection in quantum dot electroluminescent devices. *Nat. Commun.* **12**, 5669 (2021).

48. S.-K. Kim, H. Yang, Y.-S. Kim, Control of carrier injection and transport in quantum dot light emitting diodes (QLEDs) via modulating Schottky injection barrier and carrier mobility. *J. Appl. Phys.* **126**, 185702 (2019).

49. K. He, X. Zhang, S. Ren, J. Sun, Deep residual learning for image recognition, in *Proc. 29th IEEE Conf. Comput. Vis. Pattern Recognit.* 770 (IEEE, 27 to 30 June 2016).

50. M. K. Choi, J. Yang, D. C. Kim, Z. Dai, J. Kim, H. Seung, V. S. Kale, S. J. Sung, C. R. Park, N. Lu, T. Hyeon, D.-H. Kim, Extremely vivid, highly transparent, and ultrathin quantum dot light-emitting diodes. *Adv. Mater.* **30**, 1703279 (2018).

51. H. Xiao, K. Rasul, R. Vollgraf, Fashion-MNIST: A novel image dataset for benchmarking machine learning algorithms. arXiv 1708.07747 [cs.LG] (25 August 2017).

52. J. Liu, P. Liu, D. Chen, T. Shi, X. Qu, L. Chen, T. Wu, J. Ke, K. Xiong, M. Li, H. Song, W. Wei, J. Cao, J. Zhang, L. Gao, J. Tang, A near-infrared colloidal quantum dot imager with monolithically integrated readout circuitry. *Nat. Electron* **5**, 443–451 (2022)

53. T. Wang, Y. Zhang, Y. Gao, Z. Zhang, Z. Chen, D. Li, W. Mei, Y. Li, L. Zhou, C. Pei, J. Yu, H. Shi, J. Liao, X. Li, X. Xu, 63-4: Development of ink-jet printing process for 55-inch UHD AMQLED display. *SID Symposium Digest of Technical Papers* **52**, 930–932 (2021).

54. S. Choi, C.-m. Kang, C.-W. Byun, H. Cho, B.-H. Kwon, J.-H. Han, J.-H. Yang, J.-W. Shin, C.-S. Hwang, N. S. Cho, K. M. Lee, H.-O. Kim, E. Kim, S. Yoo, H. Lee, Thin-film transistor-

driven vertically stacked full-color organic light-emitting diodes for high-resolution active-matrix displays. *Nat. Commun.* **11**, 2732 (2020).

55. H. Moon, H. Seong, W. C. Shin, W.-T. Park, M. Kim, S. Lee, J. H. Bong, Y.-Y. Noh, B. J. Cho, S. Yoo, S. G. Im, Synthesis of ultrathin polymer insulating layers by initiated chemical vapour deposition for low-power soft electronics. *Nat. Mater.* **14**, 628–635 (2015).

56. M. K. Choi, J. Yang, K. Kang, D. C. Kim, C. Choi, C. Park, S. J. Kim, S. I. Chae, T.-H. Kim, J. H. Kim, T. Hyeon, D.-H. Kim, Wearable red-green-blue quantum dot light-emitting diode array using high-resolution intaglio transfer printing. *Nat. Commun.* **6**, 7149 (2015).

57. Y. Shirasaki, G. J. Supran, M. G. Bawendi, V. Bulović, Emergence of colloidal quantum-dot light-emitting technologies. *Nat. Photon.* **7**, 13–23 (2013).

58. J. Kim, H. J. Shim, J. Yang, M. K. Choi, D. C. Kim, J. Kim, T. Hyeon, D.-H. Kim, Ultrathin quantum dot display integrated with wearable electronics. *Adv. Mater.* **29**, 1700217 (2017).
